# Three types of incremental learning

**DOI:** 10.1038/s42256-022-00568-3

**Published:** 2022-12-05

**Authors:** Gido M. van de Ven, Tinne Tuytelaars, Andreas S. Tolias

**Affiliations:** 1 Center for Neuroscience and Artificial Intelligence, Department of Neuroscience, Baylor College of Medicine, Houston, TX USA; 2 Computational and Biological Learning Lab, Department of Engineering, University of Cambridge, Cambridge, UK; 3 Processing Speech and Images, Department of Electrical Engineering, KU Leuven, Leuven, Belgium; 4 Department of Electrical and Computer Engineering, Rice University, Houston, TX USA

**Keywords:** Computer science, Software, Learning algorithms

## Abstract

Incrementally learning new information from a non-stationary stream of data, referred to as ‘continual learning’, is a key feature of natural intelligence, but a challenging problem for deep neural networks. In recent years, numerous deep learning methods for continual learning have been proposed, but comparing their performances is difficult due to the lack of a common framework. To help address this, we describe three fundamental types, or ‘scenarios’, of continual learning: task-incremental, domain-incremental and class-incremental learning. Each of these scenarios has its own set of challenges. To illustrate this, we provide a comprehensive empirical comparison of currently used continual learning strategies, by performing the Split MNIST and Split CIFAR-100 protocols according to each scenario. We demonstrate substantial differences between the three scenarios in terms of difficulty and in terms of the effectiveness of different strategies. The proposed categorization aims to structure the continual learning field, by forming a key foundation for clearly defining benchmark problems.

## Abstract (web summary)

A challenge for any machine learning system is to continually adapt to new data. While methods to address this issue are developed, their performance is hard to compare. A new framework to facilitate benchmarking divides approaches into three categories, defined by whether models need to adapt to new tasks, domains or classes.

## Main

An important open problem in deep learning is enabling neural networks to incrementally learn from non-stationary streams of data^1,2^. For example, when deep neural networks are trained on samples from a new task or data distribution, they tend to rapidly lose previously acquired capabilities, a phenomenon referred to as catastrophic forgetting^3,4^. In stark contrast, humans and other animals are able to incrementally learn new skills without compromising those that were already learned^5^. The field of continual learning, also referred to as lifelong learning, is devoted to closing the gap in incremental learning ability between natural and artificial intelligence. In recent years, this area of machine learning research has been rapidly expanding, fuelled by the potential utility of deploying continual learning algorithms for applications such as medical diagnosis^6^, autonomous driving^7^ or predicting financial markets^8^.

Despite its scope, continual learning research is relatively unstructured and the field lacks a shared framework. Because of an abundance of subtle, but often important, differences between evaluation protocols, systematic comparison between continual learning algorithms is challenging, even when papers use the same datasets^9^. It is therefore not surprising that numerous continual learning methods claim to be state-of-the-art. To help address this, here we describe a structured and intuitive framework for continual learning.

We put forward the view that, at the computational level^10^, there are three fundamental types, or ‘scenarios’, of supervised continual learning. Informally, (a) in task-incremental learning, an algorithm must incrementally learn a set of clearly distinguishable tasks; (b) in domain-incremental learning, an algorithm must learn the same kind of problem but in different contexts; and (c) in class-incremental learning, an algorithm must incrementally learn to distinguish between a growing number of objects or classes. In this article, we formally define these three scenarios and point out different challenges associated with each one of them. We also review existing strategies for continual learning with deep neural networks and we provide a comprehensive, empirical comparison to test how suitable these different strategies are for each scenario.

## Three continual learning scenarios

In classical machine learning, an algorithm has access to all training data at the same time. In continual learning, the data instead arrives in a sequence, or in a number of steps, and the underlying distribution of the data changes over time. In this article, we propose that, depending on how the aspect of the data that changes over time relates to the function or mapping that must be learned, there are three fundamental ways in which a supervised learning problem can be incremental (Table 1). Below, we start by describing the resulting three continual learning scenarios intuitively. After that we define them more formally: first in a restricted, ‘academic’ setting, before generalizing them to more flexible continual learning settings.

**Table 1:** Overview of the three continual learning scenarios

Scenario	Intuitive description	Mapping to learn
**Task-incremental learning**	Sequentially learn to solve a number of distinct tasks	$\hspace{2.22144pt}f\,:{{{\mathcal{X}}}}\times {{{\mathcal{C}}}}\to {{{\mathcal{Y}}}}$
**Domain-incremental learning**	Learn to solve the same problem in different contexts	$\hspace{2.22144pt}f\,:{{{\mathcal{X}}}}\to {{{\mathcal{Y}}}}$
**Class-incremental learning**	Discriminate between incrementally observed classes	$\hspace{2.22144pt}f\,:{{{\mathcal{X}}}}\to {{{\mathcal{C}}}}\times {{{\mathcal{Y}}}}$

Notation: ${{{\mathcal{X}}}}$ is the input space, ${{{\mathcal{Y}}}}$ is the within-context output space and ${{{\mathcal{C}}}}$ is the context space. In this article, the term ‘context’ refers to an underlying distribution from which observations are sampled. The context changes over time. In the continual learning literature, the term ‘task’ is often used in a way analogous to how the term ‘context’ is used here.

### Intuitive descriptions and each scenario’s challenges

The first continual learning scenario we refer to as ‘task-incremental learning’ (or Task-IL). This scenario is best described as the case where an algorithm must incrementally learn a set of distinct tasks (see refs. ^11–13^ for examples from the literature). The defining characteristic of task-incremental learning is that it is always clear to the algorithm—also at test time—which task must be performed. In practice, this could mean that task identity is explicitly provided, or that the tasks are clearly distinguishable. In this scenario it is possible to train models with task-specific components (for example, a separate output layer per task), or even to have a completely separate network for each task to be learned. In this last case there is no forgetting at all. The challenge with task-incremental learning, therefore, is not—or should not be—to simply prevent catastrophic forgetting, but rather to find effective ways to share learned representations across tasks, to optimize the trade-off between performance and computational complexity and to use information learned in one task to improve performance on other tasks (that is, to achieve positive forward or even backward transfer between tasks)^14,15^. These are still open challenges. Real-world examples of task-incremental learning are learning to play different sports or different musical instruments, because typically it is always clear which sport or instrument should be played.

We call the second scenario ‘domain-incremental learning’ (or Domain-IL). In this scenario, the structure of the problem is always the same, but the context or input-distribution changes (for example, there are domain-shifts; see refs. ^16,17^). Similarly to task-incremental learning, this scenario can be described as that an algorithm must incrementally learn a set of ‘tasks’ (although now it might be more intuitive to think of them as ‘domains’), but with the crucial difference that—at least at test time—the algorithm does not know to which task a sample belongs. However, identifying the task is not necessary, because each task has the same possible outputs (for example, the same classes are used in each task). Using task-specific components in this scenario is, however, only possible if an algorithm first identifies the task^18–23^, but that is not necessarily the most efficient strategy. Preventing forgetting ‘by design’ is therefore not possible with domain-incremental learning, and alleviating catastrophic forgetting is still an important unsolved challenge. Examples of this scenario are incrementally learning to recognize objects under variable lighting conditions^24^ (for example, indoors versus outdoors) or learning to drive in different weather conditions^17^.

The third continual learning scenario is ‘class-incremental learning’ (or Class-IL). This scenario is best described as the case where an algorithm must incrementally learn to discriminate between a growing number of objects or classes (for example, refs. ^25,26^). An often used set-up for this scenario is that a sequence of classification-based tasks (although now it might be more intuitive to think of them as ‘episodes’) is encountered, whereby each task contains different classes and the algorithm must learn to distinguish between all classes^19,27,28^. In this case, task identification is necessary to solve the problem, as it determines which possible classes the current sample might belong to. In other words, the algorithm should be able both to solve each individual task (that is, distinguish between classes within an episode) and to identify which task a sample belongs to (that is, distinguish between classes from different episodes). For example, an agent might first learn about cats and dogs, and later about cows and horses; while with task-incremental learning the agent would not be expected to distinguish between animals encountered in different episodes (for example, between cats and cows), with class-incremental learning this is required. An important challenge in this scenario is learning to discriminate between classes that are not observed together, which has turned out to be very challenging for deep neural networks, especially when storing examples of previously seen classes is not allowed^29,30^.

### Formalization in a restricted, ‘academic’ setting

To more formally define these three scenarios, we start by considering the simple, but frequently studied, continual learning setting in which a classification problem is split up into multiple parts or episodes that must be learned sequentially, with no overlap between the different episodes. In the continual learning literature, these episodes are often called tasks, but in this article we will refer to them as ‘contexts’. The term task is problematic because in the literature it is used with several different meanings or connotations. From here on, we will use the term task only to refer to a context when it is always clear to the learning algorithm when a sample belongs to that context (as is the case with task-incremental learning).

In the ‘academic continual learning setting’ sketched above (that is, classification-based, non-overlapping contexts encountered sequentially), a clear distinction can be drawn between the three scenarios. To formalize this, we express each sample as consisting of three components: an input ${{{{x}}}}\in {{{\mathcal{X}}}}$, a within-context label $y\in {{{\mathcal{Y}}}}$ and a context label $c\in {{{\mathcal{C}}}}$. The three scenarios can then be defined based on how the function or mapping that must be learned relates to the context space ${{{\mathcal{C}}}}$. With task-incremental learning, an algorithm is expected to learn a mapping of the form $f\,:{{{\mathcal{X}}}}\times {{{\mathcal{C}}}}\to {{{\mathcal{Y}}}}$, with domain-incremental learning a mapping of the form $f\,:{{{\mathcal{X}}}}\to {{{\mathcal{Y}}}}$ must be learned and with class-incremental learning the shape of the mapping to be learned is $f\,:{{{\mathcal{X}}}}\to {{{\mathcal{C}}}}\times {{{\mathcal{Y}}}}$. For class-incremental learning this mapping can also be written as $f\,:{{{\mathcal{X}}}}\to {{{\mathcal{G}}}}$, with ${{{\mathcal{G}}}}$ the ‘global label space’ obtained by combining ${{{\mathcal{C}}}}$ and ${{{\mathcal{Y}}}}$.

**Fig. 1:**
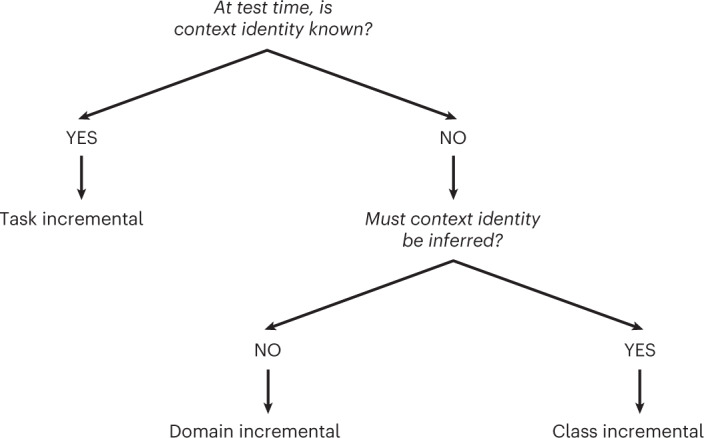
Decision tree for the three continual learning scenarios. The scenarios can be defined based on whether at test time context identity is known and if it is not, whether it must be inferred.

These definitions imply that the three scenarios can be distinguished based on whether at test time context identity information is known to the algorithm and, in case it is not, whether it must be inferred (Fig. 1). Each scenario thus specifies whether context labels are available during testing, but not necessarily whether they are available during training. With task- and class-incremental learning, it is often implicit that context labels are provided during training (for example, in the case of supervised learning), but with domain-incremental learning it is good practice to explicitly state whether context labels (or context boundaries) are provided during training.

**Fig. 2:**
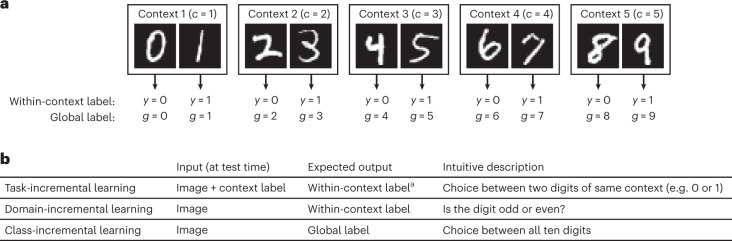
Split MNIST according to the three scenarios. **a**, The Split MNIST protocol is obtained by splitting the original MNIST dataset into five contexts, with each context consisting of two digits. **b**, Overview of what is expected of the algorithm at test time when the Split MNIST protocol is performed according to each continual learning scenario. ^a^With task-incremental learning, at the computational level, there is no difference between whether the algorithm must return the within-context label or the global label, because the within-context label can be combined with the context label (which is provided as input) to get the global label.

To illustrate the continual learning scenarios with an example, Fig. 2 shows how Split MNIST, which is a popular toy problem for continual learning^27,28,31,32^, can be performed according to each of the three scenarios. Further examples illustrating these scenarios with other context sequences are provided in Supplementary Note 1.

It might be unintuitive to distinguish domain- and class-incremental learning by whether context identity must be inferred, because with class-incremental learning context identification is often not explicitly performed, as typically a direct mapping is learned from the input space ${{{\mathcal{X}}}}$ to the set of global labels ${{{\mathcal{G}}}}$. Another way to tell these two scenarios apart is by whether different contexts contain the same classes (domain-incremental learning) or different classes (class-incremental learning). However, it should then be realized that whether two samples belong to the same class can change depending on perspective: in the Split MNIST example (Fig. 2), with domain-incremental learning the digits ‘0’ and ‘2’ belong to the same class (as they are both even digits), but with class-incremental learning they are considered different classes.

### Generalization to more flexible settings

The clear separation between the three scenarios makes the academic continual learning setting convenient for studying these scenarios and their different challenges in isolation. However, this setting does not reflect well the arbitrary non-stationarity that can be observed in the real world^33–40^. To generalize the three scenarios to more flexible continual learning settings, we first introduce a distinction between the concepts ‘context set’ and ‘data stream’:

The ‘context set’ is defined as a collection of underlying distributions, denoted by ${\left\{{{{{\mathcal{D}}}}}_{c}\right\}}_{c\in {{{\mathcal{C}}}}}$, from which the observations presented to the algorithm are sampled. For a supervised continual learning problem, for each context $c\in {{{\mathcal{C}}}}$, samples from ${{{{\mathcal{D}}}}}_{c}$ consist of an input $x\in {{{\mathcal{X}}}}$ and a within-context label $y\in {{{\mathcal{Y}}}}$. (With class-incremental learning each context could also contain a single class, in which case the within-context label *y* is not used.)

The ‘data stream’ is defined as a (possibly unbounded) stream of experiences that are sequentially presented to the algorithm: *e*_1_, *e*_2_, …. Each experience consists of a set of observations sampled from one or more of the underlying distributions of the context set. These experiences are the incremental steps of a continual learning problem, in the sense that at each step, the algorithm has free access to the data of the current experience, but not to the data from past or future experiences (see also ref. ^41^).

In the academic continual-learning setting, there is no distinction between the context set and the data stream, because each experience consists of all the training data of a particular context. In general, however, such a direct relation is not needed. Every observation within each experience can in principle be sampled from any combination of underlying datasets from the context set. This can be formalized as:

1 $${e}_{t}[i] \sim \mathop{\sum}\limits_{c\in {{{\mathcal{C}}}}}{p}_{c}^{t,i}{{{{\mathcal{D}}}}}_{c}$$

whereby *e*_*t*_[*i*] is observation *i* of experience *t* and ${p}_{c}^{t,i}$ is the probability that this observation is sampled from ${{{{\mathcal{D}}}}}_{c}$. Importantly, in this framework, from a probabilistic perspective, two observations at different points in time can only differ from each other with respect to the (combination of) contexts from which they are sampled. With this formulation, the context set describes the aspects of the data that ‘can’ change over time and the probabilities ${p}_{c}^{t,i}$ describe ‘how’ they change over time.

An advantage of distinguishing between the context set and the data stream is that it makes it possible to describe continual learning problems with gradual transitions between contexts^34,37,40,42^ or whereby contexts are revisited^43–45^. In this framework, which is suitable for so-called ‘task-free continual learning’^33,46–48^, generalized versions of the three scenarios can be defined based on how the mapping that must be learned relates to the context space ${{{\mathcal{C}}}}$, which describes the non-stationary aspect of the data. Supplementary Note 2 illustrates how a ‘task-free’ data stream can be performed according to each of the generalized versions of the three scenarios.

We note that for complex real-world incremental learning problems, it might not be straight-forward to express the mapping that must be learned in terms of the context space ${{{\mathcal{C}}}}$, for example, because there are different aspects of the data that change over time. To accommodate this, a multidimensional context space ${{{\mathcal{C}}}}$ can be used, whereby each dimension could adhere to a different scenario. This allows for continual-learning problems that are mixtures of scenarios (Supplementary Note 3). Another generalization is that contexts do not need to be discrete, but can be continuous (in that case the summation in equation (1) becomes an integral); an example of a continuous context set is Rotated MNIST with arbitrary rotation (Supplementary Note 3).

## Empirical comparison

**Fig. 3:**
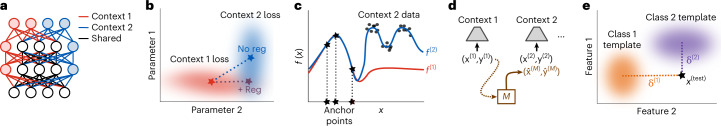
Schematic illustrations of different continual learning strategies. **a**, Context-specific components. Certain parts of the network are only used for specific contexts. **b**, Parameter regularization. Parameters important for past contexts are encouraged not to change too much when learning new contexts. **c**, Functional regularization. The input–output mapping learned previously is encouraged not to change too much at a particular set of inputs (the ‘anchor points’) when training on new contexts. **d**, Replay. The training data of a new context is complemented with data representative of past context. The replayed data is sampled from *M*, which can be a memory buffer or a generative model. **e**, Template-based classification. A ‘template’ is learned for each class (for example, a prototype, an energy value or a generative model), and classification is performed based on which template is most suitable for the sample to be classified. See Supplementary Note 4 for a detailed discussion of these strategies.

To further explore the differences between the three continual learning scenarios, here we provide an empirical comparison of the performance of different deep learning strategies. To do this comparison in a structured manner, in Supplementary Note 4 we discuss and distinguish five computational strategies for continual learning (Fig. 3). For each strategy, we included a few representative methods in our comparison.

### Compared methods

The use of context-specific components was represented by context-dependent gating (XdG^12^), which masks for each context a randomly selected subset of hidden units; and the separate networks approach, where the available parameter budget is divided over all contexts and a separate network is learned for each context.

Included parameter regularization methods were elastic weight consolidation (EWC^49^), which estimates parameter importance using a diagonal approximation to the Fisher information; and synaptic intelligence (SI^31^), which estimates parameter importance online based on the training trajectory.

For functional regularization, compared were learning without forgetting (LwF^50^), which uses the inputs from the current context as anchor points; and functional regularization of the memorable past (FROMP^51^), which uses stored examples from past contexts as anchor points.

The included replay methods were deep generative replay (DGR^27^), which replays generated representations at the input level; brain-inspired replay (BI-R^28^), which replays generated representations at the latent feature level; experience replay (ER^52,53^), which replays stored samples in the ‘standard way’ (that is, loss on replayed data added to loss on current data); and averaged gradient episodic memory (A-GEM^54^), which replays the same stored samples but using the loss on replayed data as inequality constraint (that is, loss on current data optimized under constraint that loss on replayed data cannot increase).

The compared template-based methods were iCaRL^25^, with mean latent feature representations of stored examples as templates; and the generative classifier from ref. ^55^, which uses class-specific generative models as templates.

Finally, two baselines were included. As lower target, referred to as ‘none’, the model was incrementally trained on all contexts in the standard way. As upper target, referred to as ‘joint’, the model was trained on the data of all contexts at the same time.

### Set-up

We performed both the Split MNIST and the Split CIFAR-100 protocol according to each of the three scenarios. All experiments used the academic continual learning setting and context identity information was available during training. To make the comparisons as informative as possible, we used similar network architectures and similar training protocols for all compared methods. Depending on the continual learning scenario, the output layer of the network was treated differently. With task-incremental learning, a multi-headed output layer was used whereby each context had its own output units and only the units of the context under consideration were used. For the other two scenarios, single-headed output layers were used, with the number of output units equal to the number of classes per context (domain-incremental learning) or to the total number of classes (class-incremental learning). See Methods for more detail.

### Results

For both Split MNIST (Table 2) and Split CIFAR-100 (Table 3), we found clear differences between the three continual learning scenarios. With task-incremental learning, almost all tested methods performed well compared to the ‘none’ and ‘joint’ baselines, with domain-incremental learning the relative performances of many methods dropped considerably and with class-incremental learning they decreased even further.

**Table 2:** Results on Split MNIST

Strategy	Method	Budget	GM	Task-IL	Domain-IL	Class-IL
Baselines	None – lower target			84.32 (±0.99)	60.13 (±1.66)	19.89 (±0.02)
	Joint – upper target			99.67 (±0.03)	98.59 (±0.05)	98.17 (±0.04)
Context-specific components	Separate Networks	-	-	99.57 (±0.03)	-	-
	XdG	-	-	99.10 (±0.10)	-	-
Parameter regularization	EWC	-	-	99.06 (±0.15)	63.03 (±1.58)	20.64 (±0.52)
	SI	-	-	99.20 (±0.11)	66.94 (±1.13)	21.20 (±0.57)
Functional regularization	LwF	-	-	99.60 (±0.03)	71.18 (±1.42)	21.89 (±0.32)
	FROMP	100	-	99.12 (±0.13)	84.86 (±1.02)	77.38 (±0.64)
Replay	DGR	-	Yes	99.50 (±0.03)	95.57 (±0.30)	90.35 (±0.24)
	BI-R	-	Yes	99.61 (±0.03)	97.26 (±0.15)	94.41 (±0.15)
	ER	100	-	98.98 (±0.07)	93.75 (±0.24)	88.79 (±0.20)
	A-GEM	100	-	98.54 (±0.10)	87.67 (±1.33)	65.10 (±3.64)
Template-based classification	Generative Classifier	-	Yes	-	-	93.82 (±0.06)
	iCaRL	100	-	-	-	92.49 (±0.12)

Reported is the final test accuracy (as percentage, averaged over all contexts) of all compared methods on the Split MNIST protocol, which is performed according to all three scenarios. The experiments followed the academic continual learning setting and context identity information was available during training. The column ‘Budget’ indicates the number of examples per class that was allowed to be stored in a memory buffer. The column ‘GM’ indicates whether a generative model was learned, for which additional network capacity was used. Each experiment was performed 20 times with different random seeds, reported is the mean (±s.e.m.) over these runs.

**Table 3:** Results on Split CIFAR-100

Strategy	Method	Budget	GM	Task-IL	Domain-IL	Class-IL
Baselines	None – lower target			61.43 (±0.36)	18.42 (±0.33)	7.71 (±0.18)
	Joint – upper target			78.78 (±0.25)	46.85 (±0.51)	49.78 (±0.21)
Context-specific components	Separate Networks	-	-	76.83 (±0.25)	-	-
	XdG	-	-	69.86 (±0.34)	-	-
Parameter regularization	EWC	-	-	76.34 (±0.29)	21.65 (±0.55)	8.24 (±0.25)
	SI	-	-	74.84 (±0.39)	22.58 (±0.42)	8.10 (±0.24)
Functional regularization	LwF	-	-	78.59 (±0.24)	29.45 (±0.39)	25.57 (±0.27)
Replay	DGR	-	Yes	71.40 (±0.32)	20.52 (±0.43)	9.67 (±0.22)
	BI-R	-	Yes	79.14 (±0.21)	30.26 (±0.44)	25.81 (±0.41)
	ER	100	-	76.43 (±0.24)	39.00 (±0.34)	37.57 (±0.21)
	A-GEM	100	-	73.30 (±0.39)	20.51 (±0.59)	20.38 (±1.45)
Template-based classification	Generative Classifier	-	Yes	-	-	46.83 (±0.18)
	iCaRL	100	-	-	-	37.83 (±0.21)

Reported is the final test accuracy (as percentage, averaged over all contexts) of all compared methods on the Split CIFAR-100 protocol, which is performed according to all three scenarios. The experiments followed the academic continual learning setting and context identity information was available during training. The column ‘Budget’ indicates the number of examples per class that was allowed to be stored in a memory buffer. The column ‘GM’ indicates whether a generative model was learned, for which additional network capacity was used. Note that we were not able to run the method FROMP on this protocol due to its high computational costs. Each experiment was performed 10 times with different random seeds, reported is the mean (±s.e.m.) over these runs. All compared methods used convolutional layers that were pre-trained on CIFAR-10, see Methods for full details.

The decline in performance across the three scenarios was most pronounced for the parameter regularization methods. On both protocols, EWC and SI performed close to the upper target when context identity was known during testing (that is, task-incremental learning); with domain-incremental learning the performance of both methods was substantially lower, but remained above the lower target of sequentially training a network in the standard way; and with class-incremental learning the performance of EWC and SI was similar to the lower target, indicating that in this scenario these methods failed completely. There was a similar trend across the three scenarios for the functional regularization methods, albeit less pronounced for FROMP than for LwF.

Replay-based methods performed relatively well in all three scenarios. Although on both protocols their performance still decreased from task- to domain- to class-incremental learning, the decline was less sharp than for the regularization-based methods, and replay-based methods were among the top performers in each scenario. Template-based classification also performed well with class-incremental learning, with iCaRL and the Generative Classifier among the best performing methods on both protocols.

For class-incremental learning, the methods that performed best either used a generative model or they stored previously seen data in a memory buffer. Directly comparing methods using these two types of memories can be arbitrary, as their performance can heavily depend on the number of stored examples or the kind of generative model. We instead focus on comparing methods using the same type of memory.

For methods using generative models, the largest differences were observed with Split CIFAR-100. In particular, DGR did not perform well on this protocol, indicating that standard generative replay (that is, at the input level) is not a good approach when the input data are complex (see also refs. ^28,56,57^). There was also a substantial gap in performance between BI-R and the Generative Classifier. As both methods had a generative model on the latent features, this suggests that the way in which a generative model is used (that is, for generating replay or as templates) is important as well.

**Extended Data Fig. 1:**
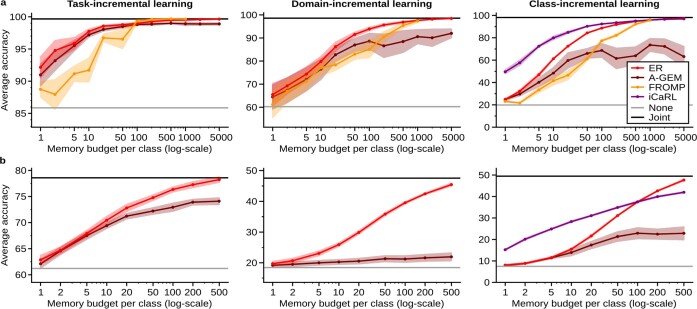
Comparison of methods using stored data with different buffer sizes. Shown is the average test accuracy (as %, over all contexts) of different methods that use stored data on Split MNIST (**a**) and on Split CIFAR-100 (**b**) as a function of the number of examples per class that is allowed to be stored in memory. Due to its high computational costs, we were not able to run FROMP on Split CIFAR-100, or on Split MNIST with a memory budget above 1,000 samples per class. Displayed are the means over 5 repetitions, shaded areas are ± 1 SEM. ER: exact replay, A-GEM: averaged gradient episodic memory, FROMP: functional regularization of the memorable past, iCaRL: incremental classifier and representation learning, None: sequential training in the standard way, Joint: training on all data at the same time.

For methods using stored data, we found that replaying stored data in the standard way (as is done by ER) was not often outperformed by more complex ways of using stored data. In fact, perhaps surprisingly, on all experiments ER comfortably outperformed A-GEM, and ER performed significantly better than FROMP on two of the three scenarios of Split MNIST. These results held for different sizes of the memory buffer (Extended Data Fig. 1). A clear improvement over ER was only observed with iCaRL, and only when the size of the memory buffer was relatively small.

## Discussion

Continual learning is a key feature of natural intelligence, but an open challenge for deep learning. Standard deep neural networks tend to catastrophically forget previous tasks or data distributions when trained on a new one. Enabling these networks to incrementally learn, and retain, information from different contexts has become a topic of intense research. Yet, despite its scope, the continual learning field lacks structure and direct comparisons between published papers can be misleading. Here, we pointed out that an important difference between continual learning set-ups is whether context identity is known to the algorithm and—if it is not—whether it must be inferred. Based on these two distinctions, we identified three scenarios for continual learning: task-incremental learning, domain-incremental learning and class-incremental learning.

These three scenarios and their different challenges can be conveniently studied in an academic continual learning setting, where a classification-based problem is split up in discrete, non-overlapping contexts (which are often called ‘tasks’) that are encountered in sequence. We showed that in this setting there is a clear separation between the three scenarios. At least in part because of two preprints of this article^58,59^, the terms ‘task-incremental learning’, ‘domain-incremental learning’ and ‘class-incremental learning’ are sometimes being used in the recent literature in a way that restricts them to this academic setting. Here, by interpreting these three scenarios as specifying how the non-stationary aspect of the data relates to the mapping that must be learned, we propose that they generalize to more flexible continual-learning settings. To demonstrate the value of such generalized versions of these three scenarios, Supplementary Note 2 shows how a ‘task-free’ data stream without sharp context boundaries can also be performed in three different ways.

A key insight of this article is that a useful way to categorize continual-learning problems is based on how the non-stationary aspect of the data relates to the mapping to be learned. For supervised classification this leads to the three continual learning scenarios discussed here, but the same perspective might also be useful for unsupervised or reinforcement learning (Supplementary Note 5). Continual learning can be categorized in other ways as well, some of which we discuss in Supplementary Note 6.

Using the academic continual-learning setting, for each scenario we performed an empirical comparison of a representative selection of continual learning algorithms. This comparison revealed marked differences between the three scenarios in overall difficulty level and in the relative effectiveness of different continual learning strategies. The only strategy among the top performers in all three scenarios is replay, with the replayed data sampled either from a memory buffer or a generative model. Surprisingly, within the class of methods using stored data, the strongest performance is often obtained by the method ER, which replays stored data ‘in the standard way’. In our experiments, popular methods such as A-GEM and FROMP, which use stored data in more complex ways, are almost always outperformed by ER, even though the computational costs of A-GEM and FROMP are strictly higher than those of ER.

In the class-incremental learning scenario, we found that parameter regularization methods such as EWC and SI fail almost completely, even on Split MNIST. Functional regularization typically works better, especially when using stored data as anchor points, but this strategy also does not work optimally. We hypothesize that regularization-based strategies—at least by themselves—are not well suited for class-incremental learning because they do not provide a way to compare between classes that are not observed together. Regularization-based methods aim to learn new contexts while preserving the function or parameters learned in previous contexts. However, with class-incremental learning, learning a new context (for example, distinguishing ‘2’ and ‘3’) while preserving what was learned before (for example, distinguishing ‘0’ and ‘1’) is not enough; it is also needed to combine information from different contexts (for example, for distinguishing ‘1’ and ‘2’). For learning to distinguish between classes not observed together, it might be unavoidable to use either replay, which allows for comparing between classes from different contexts during training, or template-based classification, which allows for comparing between classes from different contexts during inference (that is, during the classification decision).

Task-incremental learning is sometimes considered ‘easy’, and it has been argued that the continual-learning community should move away from the assumption that context identities (or task labels, as they are often called) are provided at test time^60^. A reason for this notion might be that with task-incremental learning, the bar is often set too low. In our experiments, while all methods indeed perform substantially better than the usual ‘lower target’ in which a single shared neural network is sequentially trained on all contexts, most methods perform worse than the more appropriate lower target in which a smaller, separate network is trained for each context. To do better than this ‘Separate Networks’ approach, positive forward or backward transfer between contexts is necessary, but achieving such positive transfer is not trivial^14,15^.

Domain-incremental learning might be the least studied continual learning scenario. A few years ago this scenario was regularly studied with Permuted MNIST^31,49,61^, but this protocol is not often used anymore as it is considered too artificial. The continual learning field is currently dominated by context sets created by splitting up existing image classification datasets based on class labels (for example, Split MNIST, Split CIFAR-100). Although in theory these context sets can be performed according to all three scenarios, they are typically less intuitive and/or realistic under the assumptions of domain-incremental learning. However, in recent years, several resources have been created that provide—or enable the generation of—more realistic context sets well suited for domain-incremental learning^24,41,62–64^. These resources might help to renew the community’s interest in this scenario.

The three continual learning scenarios described in this article provide a useful basis for defining clear and unambiguous benchmark problems for continual learning. We hope this will accelerate progress to bridge the gap between natural and artificial intelligence. Moreover, we believe that it is an important conceptual insight that, at the computational level, a supervised learning problem can be incremental in these three different ways. Perhaps especially in the real world, where continual learning problems are often complex and ‘mixtures’ of scenarios, it might be fruitful to approach problems as consisting of a combination of these three fundamental types of incremental learning.

## Methods

All experiments were run using custom-written code for the Python machine learning framework PyTorch^65^.

### Context sets

For the Split MNIST protocol, the MNIST dataset^66^ was split into five contexts, such that each context contained two digits. The digits were randomly divided over the five contexts, so the order of the digits was different for each random seed. The original 28×28 pixel greyscale images were used without pre-processing. The standard training/test-split was used, which resulted in 60,000 training images (approximately 6,000 per digit) and 10,000 test images (approximately 1,000 per digit).

For the Split CIFAR-100 protocol, the CIFAR-100 dataset^67^ was split up into ten contexts, such that each context contained ten image classes. The classes were randomly divided over the contexts, with a different class order for each random seed. The original 32×32 pixel RGB-colour images were normalized (that is, each pixel-value was subtracted by the relevant channel-wise mean and divided by the channel-wise standard deviation, with means and standard deviations calculated over all training images). No other pre-processing or augmentation was applied. The standard training/test-split was used, which resulted in 50,000 training images (500 per class) and 10,000 test images (100 per class).

### Base neural network architecture

To make the comparisons as informative as possible, we used the same base neural network architecture for all methods as much as possible. For Split MNIST, the base network had two fully connected hidden layers of 400 ReLU each and a softmax output layer. For Split CIFAR-100, the base network had five pre-trained convolutional layers followed by two fully connected layers with 2,000 ReLU each and a softmax output layer. The convolutional layers contained 16, 32, 64, 128 and 256 channels. Each convolutional layer used a 3×3 kernel, a padding of 1 and there was a stride of 1 in the first layer (that is, no downsampling) and a stride of 2 in the other layers (that is, image-size was halved in each of those layers). Batch norm^68^ was used in all convolutional layers, followed by a ReLU non-linearity. No pooling was used. The convolutional layers were pre-trained on CIFAR-10, which is a dataset containing similar but non-overlapping images and image classes compared with CIFAR-100^67^. To pre-train the convolutional layers, the base neural network was trained to classify the 10 classes of CIFAR-10 for 100 epochs, using the ADAM-optimizer (*β*_1_ = 0.9, *β*_2_ = 0.999) with learning rate of 0.0001 and mini-batch size of 256. For the pre-training on CIFAR-10, images were normalized and augmented by random cropping and horizontal flipping. A similar pre-training protocol was used in ref. ^28^. During the incremental training on CIFAR-100, the parameters of the pre-trained convolutional layers were frozen. For all compared methods, freezing these parameters resulted in similar or better performance compared with not freezing them.

### Output layer

The softmax output layer of the network was treated differently depending on the continual learning scenario that was performed. With task-incremental learning, a multi-headed output layer was used, meaning that each context had its own output units and only the output units of the context under consideration—that is, either the current context or the replayed context—were set to ‘active’ (see next paragraph). With domain- and class-incremental learning, a single-headed output layer was used. For domain-incremental learning, this meant that all contexts used the same output units (that is, there were 2 output units for Split MNIST and 10 for Split CIFAR-100); for class-incremental learning, this meant that each class had its own output unit (that is, there were 10 output units for Split MNIST and 100 for Split CIFAR-100). With both domain- and class-incremental learning, always all output units were set to ‘active’. Note that with class-incremental learning another possibility is to use an ‘expanding head’ and only set the output units of classes seen so far to active (for example, see refs. ^28,69^). We found that for our experiments there was not much difference in performance between these two options. Because all output units should always be active for the Bayesian interpretation of the parameter regularization methods^21^, we decided to use that approach in this study.

Whether an output unit was set to ‘active’ controlled whether a network could assign a positive probability to its corresponding class. The probability predicted by a neural network with parameters *θ* that an input *x* belongs to output class *o* was calculated as:

2 $${p}_{{{{{\theta }}}}}\left(o| {{{{x}}}}\right)=\left\{\begin{array}{ll}\frac{{e}^{{z}_{o}^{({{{{x}}}},{{{{\theta }}}})}}}{{\sum }_{j}{e}^{{z}_{j}^{({{{{x}}}},{{{{\theta }}}})}}}\quad &{{{\rm{if}}}}\,{{{\rm{output}}}}\,{{{\rm{unit}}}}\,o\,{{{\rm{is}}}}\,{{{\rm{active}}}}\\ {{{\rm{0}}}}\quad &{{{\rm{otherwise}}}}\end{array}\right.$$

whereby ${z}_{o}^{({{{{x}}}},{{{{\theta }}}})}$ was the logit of output class *o* obtained by putting input *x* through the neural network with parameters *θ*. The summation in the denominator was over all active classes in the output layer. Importantly, with task- and class-incremental learning, output class *o* refers to the ‘global class’ that is obtained by combining the within-context label *y* and the context label *c* (that is, the set of global classes is given by ${{{\mathcal{G}}}}={{{\mathcal{Y}}}}\times {{{\mathcal{C}}}}$). With domain-incremental learning, output class *o* refers to the within-context label *y*.

### Data stream

All experiments in this article used the academic continual learning setting, meaning that the different contexts were presented to the algorithm one after the other. Within each context, the training data was fed to the algorithm in a stream of independent and identically distributed experiences (or iterations). For Split MNIST, each context was trained for 2,000 iterations with mini-batch size of 128. For Split CIFAR-100, there were 5,000 iterations per context with mini-batch size of 256. Some of the compared methods (EWC, FROMP and iCaRL) performed an additional pass over each context’s training data upon finishing training on that context.

### Loss function and optimization

For all compared methods, the parameters of the neural network were sequentially trained on each context by optimizing a loss function (denoted by ${{{{\mathcal{L}}}}}_{{{{\rm{total}}}}}$) using stochastic gradient descent. In each iteration, the loss was calculated as the average over all samples in the mini-batch and a single gradient step was taken with the ADAM-optimizer (*β*_1_ = 0.9, *β*_2_ = 0.999; ref. ^70^) and a learning rate of either 0.001 (Split MNIST) or 0.0001 (Split CIFAR-100).

For most compared methods, a central component of the loss function was the multi-class cross-entropy classification loss on the data of the current context. For an input *x* labeled with a hard target *o*, this classification loss was given by:

3 $${{{{\mathcal{L}}}}}^{{{{\rm{C}}}}}\left({{{{x}}}},o;{{{{\theta }}}}\right)=-\log {p}_{{{{{\theta }}}}}\left(o| {{{{x}}}}\right)$$

with *p*_***θ***_ the conditional probability distribution defined by the neural network with parameters *θ*, as given in equation (2).

### Memory buffer and generative models

Several of the compared methods (FROMP, ER, A-GEM and iCaRL) maintained a memory buffer in which examples of previously seen classes were stored. Except for the experiments in Extended Data Fig. 1, 100 examples per class were allowed to be stored in the memory buffer (that is, the per-class memory budget *B* was set to 100). Some other methods (DGR, BI-R and the Generative Classifier) learned generative models, these methods used up to three times as many parameters compared with the other methods.

### Baselines

For the baseline ‘None’, which was included as a lower target, the base neural network was sequentially trained on each context in the standard way, meaning that the loss function to be optimized was always just the classification loss on the current data (that is, ${{{{\mathcal{L}}}}}_{{{{\rm{total}}}}}={{{{\mathcal{L}}}}}^{{{{\rm{C}}}}}$).

For the baseline ‘Joint’, which was included as an upper target, the base neural network was trained on the data from all contexts at the same time. For this baseline, the same total number of iterations was used as with the sequential training protocol (that is, 5×2,000 iterations for Split MNIST and 10×5,000 iterations for Split CIFAR), but each mini-batch was always sampled jointly from the data of all contexts.

### Approaches using context-specific components

For XdG and the ‘Separate Networks’ approach, not all parts of the network were used for each context. These approaches require knowledge of which context a sample belongs to (to select the correct context-specific components), which meant that they could only be used in the task-incremental learning scenario. For both approaches, training was performed using just the classification loss on the current data (that is, ${{{{\mathcal{L}}}}}_{{{{\rm{total}}}}}={{{{\mathcal{L}}}}}^{{{{\rm{C}}}}}$).

In the task-incremental learning scenario, the other methods (that is, all methods except XdG and Separate Networks) used the available context identity information only in the form of a separate output layer for each context. This is a common and often sensible way to use context identity information, although in Supplementary Note 7 we show that sometimes it is more efficient to use context identity information in other ways.

#### Separate Networks

For the Separate Networks approach, the available parameter budget was equally divided over all contexts to be learned, and a separate sub-network was trained for each context. For Split MNIST, each context-specific sub-network had two fully connected hidden layers of 100 ReLU each and a softmax output layer. For Split CIFAR-100, the pre-trained and frozen convolutional layers were shared between all contexts, and only the fully connected part of the network was split up into context-specific sub-networks. Each context-specific sub-network had two fully connected layers with 400 ReLU each and a softmax output layer.

#### XdG

With XdG^12^, the base neural network was used and for each context a different, randomly selected subset of *X*% of the units in each hidden layer was fully gated (that is, their activations were set to zero), with *X* a hyperparameter whose value was set by a grid search (Supplementary Note 8).

### Parameter regularization methods

For the parameter regularization methods EWC and SI, a regularization term was added to the classification loss: ${{{{\mathcal{L}}}}}_{{{{\rm{total}}}}}={{{{\mathcal{L}}}}}^{{{{\rm{C}}}}}+{{{{\mathcal{L}}}}}_{{{{\rm{param-reg}}}}}$. This regularization term penalized changes to parameters thought to be important for previously learned contexts.

#### EWC

The regularization term of EWC^49^ consisted of a quadratic penalty term for each previously learned context, whereby the term of each context penalized parameters for how different they were compared to their value directly after finishing training on that context. When training on context *K* > 1, the EWC regularization term was given by:

4 $${{{{\mathcal{L}}}}}_{{{{\rm{param-reg}}}}}^{(K)}\left({{{{\theta }}}}\right)=\lambda \mathop{\sum }\limits_{k=1}^{K-1}\left(\frac{1}{2}\mathop{\sum }\limits_{i=1}^{{N}_{{{{\rm{params}}}}}}{F}_{ii}^{(k)}{\left({\theta }_{i}-{\hat{\theta }}_{i}^{(k)}\right)}^{2}\right)$$

with *λ* a hyperparameter controlling the regularization strength (which was set based on a grid search, Supplementary Note 8), ${\hat{\theta }}_{i}^{(k)}$ the value of the *i*^th^ parameter at the end of training on context *k*, and ${F}_{ii}^{(k)}$ the estimated importance of parameter *i* for context *k*. This importance estimate was calculated as the *i*^th^ diagonal element of the Fisher information matrix of context *k*:

5 $${F}_{ii}^{(k)}=\frac{1}{| {S}^{(k)}| }\mathop{\sum}\limits_{{{{{x}}}}\in {S}^{(k)}}\left(\mathop{\sum}\limits_{o}{\hat{o}}_{k}^{\left({{{{x}}}}\right)}{\left({\left.\frac{\delta \log {p}_{{{{{\theta }}}}}\left(o| {{{{x}}}}\right)}{\delta {\theta }_{i}}\right|}_{{{{{\theta }}}} = {\hat{{{{{\theta }}}}}}^{(k)}}\right)}^{2}\right)$$

whereby *S*^(*k*)^ was the training data of context *k* and ${\hat{o}}_{k}^{\left({{{{x}}}}\right)}$ was the probability that *x* belongs to output class *o*, as predicted by the network after finishing training on context *k*—that is, ${\hat{o}}_{k}^{\left({{{{x}}}}\right)}={p}_{{\hat{{{{{\theta }}}}}}^{(k)}}\left(o| {{{{x}}}}\right)$. The inner summation in equation (5) was over all output classes that were active during training on context *k*.

#### SI

The regularization term of SI (ref. ^31^) consisted of a single quadratic term that penalized changes to the parameters away from the value they had after finishing training on the previous context. When training on context *K* > 1, the SI regularization term was given by:

6 $${{{{\mathcal{L}}}}}_{{{{\rm{param-reg}}}}}^{(K)}\left({{{{\theta }}}}\right)=\gamma \mathop{\sum }\limits_{i=1}^{{N}_{{{{\rm{params}}}}}}{{{\Omega }}}_{i}^{(K-1)}{\left({\theta }_{i}-{\hat{\theta }}_{i}^{* }\right)}^{2}$$

with *γ* a hyperparameter controlling the regularization strength (which was set based on a grid search, see Supplementary Note 8), ${\hat{\theta }}_{i}^{* }$ the value of the *i*^th^ parameter at the end of training on context *K* − 1, and ${{{\Omega }}}_{i}^{(K-1)}$ the estimated importance of parameter *i* after the first *K* − 1 contexts have been learned. To compute these parameter importance estimates, after each context *k*, a per-parameter contribution to the change of the loss was calculated for each parameter *i* as follows:

7 $${\omega }_{i}^{(k)}=\mathop{\sum }\limits_{t=1}^{{N}_{{{{\rm{iters}}}}}}\left({\theta }_{i}[{t}^{(k)}]-{\theta }_{i}[{\left(t-1\right)}^{(k)}]\right)\frac{-\delta {{{{\mathcal{L}}}}}_{{{{\rm{total}}}}}[{t}^{(k)}]}{\delta {\theta }_{i}}$$

with *N*_iters_ the number of iterations per context, *θ*_*i*_[*t*^(*k*)^] the value of parameter *i* after the *t*^th^ training iteration on context *k* and $\frac{\delta {{{{\mathcal{L}}}}}_{{{{\rm{total}}}}}[{t}^{(k)}]}{\delta {\theta }_{i}}$ the gradient of the loss with respect to parameter *i* during the *t*^th^ training iteration on context *k*. For every context, these per-parameter contributions were normalized by the square of the total change of that parameter during training on that context plus a small dampening term *ξ* (set to 0.1, to bound the resulting normalized contributions when a parameter’s total change goes to zero), after which they were summed over all contexts so far:

8 $${{{\Omega }}}_{i}^{(K-1)}=\mathop{\sum }\limits_{k=1}^{K-1}\frac{{\omega }_{i}^{(k)}}{{\left({{{\Delta }}}_{i}^{(k)}\right)}^{2}+\xi }$$

with ${{{\Delta }}}_{i}^{(k)}={\theta }_{i}[{{N}_{{{{\rm{iters}}}}}}^{(k)}]-{\theta }_{i}[{0}^{(k)}]$, where *θ*_*i*_[0^(*k*)^] was the value of parameter *i* right before starting training on context *k*.

### Functional regularization methods

Similar as with parameter regularization, the functional regularization methods LwF and FROMP had a regularization term added to the classification loss: ${{{{\mathcal{L}}}}}_{{{{\rm{total}}}}}={{{{\mathcal{L}}}}}^{{{{\rm{C}}}}}+{{{{\mathcal{L}}}}}_{{{{\rm{func-reg}}}}}$. This regularization term encouraged the input–output mapping of the network not to change too much at a set of anchor points.

#### LwF

The method LwF (ref. ^50^) used the inputs from the current context as anchor points in combination with knowledge distillation^71^. During training on context *K* > 1, the LwF regularization term was given by:

9 $${{{{\mathcal{L}}}}}_{{{{\rm{func-reg}}}}}^{(K)}\left({{{{x}}}},{{{{\theta }}}}\right)=-\mathop{\sum }\limits_{k=1}^{K-1}\mathop{\sum}\limits_{o\in {{{{\mathcal{O}}}}}_{k}}{p}_{{\hat{{{{{\theta }}}}}}^{* }}^{T}\left(o| {{{{x}}}}\right)\log \left[{p}_{{{{{\theta }}}}}^{T}\left(o| {{{{x}}}}\right)\right]$$

whereby ${{{{\mathcal{O}}}}}_{k}$ was the set of output classes in context *k*, ${\hat{{{{{\theta }}}}}}^{* }$ was the parameter vector with values as they were at the end of training on context *K* − 1 and ${p}_{{{{{\theta }}}}}^{T}\left(o| {{{{x}}}}\right)$ was the ‘temperature-raised’ probability that input ***x*** belongs to output class *o*, as predicted by the network with parameters *θ*. These temperature-raised probabilities were defined as:

10 $${p}_{{{{{\theta }}}}}^{T}\left(o| {{{{x}}}}\right)=\frac{\exp \left[{z}_{o}^{({{{{x}}}},{{{{\theta }}}})}/T\right]}{{\sum }_{j}\exp \left[{z}_{j}^{({{{{x}}}},{{{{\theta }}}})}/T\right]}$$

with *T* the temperature, which was set to 2, and ${z}_{o}^{({{{{x}}}},{{{{\theta }}}})}$ the logit of output class *o* obtained by putting input ***x*** through the neural network with parameters *θ*. The summation in the denominator was over all active classes in the output layer. With task-incremental learning, for each context’s term in the outer summation of equation (9), only the output classes contained in that context were active. With domain- and class-incremental learning, always all output classes were active. In each iteration, the LwF regularization term was computed as average over the same inputs that were used to compute ${{{{\mathcal{L}}}}}^{{{{\rm{C}}}}}$.

We note that this implementation of LwF differs slightly from the implementation of LwF used in ref. ^28^. Compared with that implementation, the regularization term here was weighted less strongly, which substantially improved the performance of LwF on Split CIFAR-100. Initial experiments indicated that by reducing the weight of the replay term in equation (16) it is also be possible to improve the performance of several of the replay methods on Split CIFAR-100, but at the cost of impaired performance on Split MNIST.

#### FROMP

The method FROMP (ref. ^51^) performed functional regularization in a Bayesian framework and used stored data from previous contexts, referred to as memorable inputs, as anchor points. During training on context *K*, the regularization term of FROMP was given by:

11 $${{{{\mathcal{L}}}}}_{{{{\rm{func-reg}}}}}^{(K)}\left({{{{\theta }}}}\right)=\frac{1}{2}\tau \mathop{\sum }\limits_{k=1}^{K-1}\mathop{\sum}\limits_{o\in {{{{\mathcal{O}}}}}_{k}}{\left({{{{{m}}}}}_{k,o}^{({{{{\theta }}}})}-{{{{{m}}}}}_{k,o}^{({\hat{{{{{\theta }}}}}}^{* })}\right)}^{T}{{{{{\bf{K}}}}}_{k,o}^{(K-1)}}^{-1}\left({{{{{m}}}}}_{k,o}^{({{{{\theta }}}})}-{{{{{m}}}}}_{k,o}^{({\hat{{{{{\theta }}}}}}^{* })}\right)$$

with *τ* a hyperparameter controlling the regularization strength (which was set based on a grid search, see Supplementary Note 8) and ${\hat{{{{{\theta }}}}}}^{* }$ the parameter vector with values as they were at the end of training on context *K* − 1. Further, ${{{{{m}}}}}_{k,o}^{\left({{{{\theta }}}}\right)}$ was a vector containing for each memorable input from context *k* the probability that this input belongs to output class *o* as predicted by the network with parameters *θ*. That is, the *i*^th^ element of ${{{{{m}}}}}_{k,o}^{\left({{{{\theta }}}}\right)}$ was given by ${{{{{m}}}}}_{k,o}^{\left({{{{\theta }}}}\right)}[i]={p}_{{{{{\theta }}}}}\left(o| {{{{{x}}}}}^{(i,k)}\right)$, with *x*^(*i*,*k*)^ the *i*^th^ memorable input of context *k*. Finally, ${{{{\bf{K}}}}}_{k,o}^{(K)}$ was a matrix whose elements were given by:

12 $${{{{\bf{K}}}}}_{k,o}^{(K)}[i,j]={{{{{g}}}}}_{k,o}[i]{{{{\bf{V}}}}}^{(K)}{{{{{{g}}}}}_{k,o}[j]}^{T}$$

with:

13 $${{{{{g}}}}}_{k,o}[i]={\left.\frac{\delta {p}_{{{{{\theta }}}}}\left(o| {{{{{x}}}}}^{(i,k)}\right)}{\delta {{{{\theta }}}}}\right|}_{{{{{\theta }}}} = {\hat{{{{{\theta }}}}}}^{* }}$$

and **V**^(*K*)^ was a diagonal matrix with diagonal *v*^(*K*)^ given by:

14 $$\frac{1}{{{{{{v}}}}}^{(K)}}=\mathop{\sum }\limits_{k=1}^{K}\mathop{\sum}\limits_{{{{{x}}}}\in {{{{\mathcal{D}}}}}_{k}}{{{\rm{diag}}}}\left({{{{{\bf{J}}}}}^{(k)}({{{{x}}}})}^{T}{{{{{\Lambda }}}}}^{(k)}({{{{x}}}}){{{{\bf{J}}}}}^{(k)}({{{{x}}}})\right)$$

whereby ${{{{\bf{J}}}}}^{(k)}({{{{x}}}})={\left.\frac{\delta {{{{{f}}}}}_{{{{{\theta }}}}}({{{{x}}}})}{\delta {{{{\theta }}}}}\right|}_{{{{{\theta }}}} = {\hat{{{{{\theta }}}}}}^{(k)}}$, with *f*_*θ*_(*x*) the logits obtained by putting input *x* through the neural network with parameters *θ*, and ${{{{{\Lambda }}}}}^{(k)}({{{{x}}}})[i,j]={p}_{{{{{{\theta }}}}}^{(k)}}\left(i| {{{{x}}}}\right)\left(1-{p}_{{{{{{\theta }}}}}^{(k)}}\left(j| {{{{x}}}}\right)\right).$

The selection of memorable inputs, which are FROMP’s anchor points, took place after finishing training on each context. After finishing on context *k*, for each input *x* in that context’s training set, a relevance score was calculated as:

15 $$r\left({{{{x}}}}\right)=\mathop{\sum}\limits_{o\in {{{{\mathcal{O}}}}}_{k}}{p}_{{{{{\theta }}}}}\left(o| {{{{x}}}}\right)\left(1-{p}_{{{{{\theta }}}}}\left(o| {{{{x}}}}\right)\right)$$

whereby ${{{{\mathcal{O}}}}}_{k}$ was the set of output classes in context *k* and *θ* were the parameters after training on context *k*. Then, for each output class in context *k*, the *B* inputs with the highest relevance scores were selected as the memorable inputs for that class and stored in the memory buffer.

### Replay-based methods

The replay-based methods had two separate loss terms: one for the data of the current context, denoted as ${{{{\mathcal{L}}}}}_{{{{\rm{current}}}}}$, and one for the replayed data, denoted as ${{{{\mathcal{L}}}}}_{{{{\rm{replay}}}}}$. Except with A-GEM, during training the objective was to optimize an overall loss function that was a weighted sum of these two terms, with the weights depending on how many contexts had been seen so far:

16 $${{{{\mathcal{L}}}}}_{{{{\rm{total}}}}}=\frac{1}{{N}_{{{{\rm{contexts}}}}\,{{{\rm{so}}}}\,{{{\rm{far}}}}}}{{{{\mathcal{L}}}}}_{{{{\rm{current}}}}}+(1-\frac{1}{{N}_{{{{\rm{contexts}}}}\,{{{\rm{so}}}}\,{{{\rm{far}}}}}}){{{{\mathcal{L}}}}}_{{{{\rm{replay}}}}}$$

In each iteration, the number of replayed samples was always equal to the number of samples from the current context (that is, 128 for Split MNIST and 256 for Split CIFAR-100).

#### ER

With ER, the term ${{{{\mathcal{L}}}}}_{{{{\rm{current}}}}}$ was the standard classification loss on the data of the current context (that is, ${{{{\mathcal{L}}}}}_{{{{\rm{current}}}}}={{{{\mathcal{L}}}}}^{{{{\rm{C}}}}}$). The term ${{{{\mathcal{L}}}}}_{{{{\rm{replay}}}}}$ was also the standard classification loss, but on the replayed data. In each iteration, the samples to be replayed were randomly sampled from the memory buffer. The memory buffer was updated after each context, when for each new class *B* samples were randomly selected from the training data and added to the buffer.

#### A-GEM

For the method A-GEM (ref. ^54^), the loss terms ${{{{\mathcal{L}}}}}_{{{{\rm{current}}}}}$ and ${{{{\mathcal{L}}}}}_{{{{\rm{replay}}}}}$ were defined similarly as for ER. The population of the memory buffer and sampling of the the data to be replayed from the memory buffer were also the same. The only difference compared to ER was that with A-GEM, the objective was not to minimize the combined loss (that is, ${{{{\mathcal{L}}}}}_{{{{\rm{total}}}}}$), but instead the objective was to minimize the loss on the current data (that is, ${{{{\mathcal{L}}}}}_{{{{\rm{current}}}}}$) under the constraint that the loss on the replayed data (that is, ${{{{\mathcal{L}}}}}_{{{{\rm{replay}}}}}$) did not increase. To achieve this, in every iteration, the gradient vector that was used to update the parameters (that is, the gradient vector that was put into the ADAM-optimizer) was required to have a positive angle with the gradient of ${{{{\mathcal{L}}}}}_{{{{\rm{replay}}}}}$. Therefore, whenever the angle between the gradient of ${{{{\mathcal{L}}}}}_{{{{\rm{current}}}}}$ and the gradient of ${{{{\mathcal{L}}}}}_{{{{\rm{replay}}}}}$ was negative, the gradient of ${{{{\mathcal{L}}}}}_{{{{\rm{current}}}}}$ was projected onto the orthogonal complement of the gradient of ${{{{\mathcal{L}}}}}_{{{{\rm{replay}}}}}$. Let ${{{{\mathcal{B}}}}}_{{{{\rm{current}}}}}$ be the mini-batch of data from the current context and ${{{{\mathcal{B}}}}}_{{{{\rm{replay}}}}}$ the mini-batch of replayed data from the memory buffer. The gradient of ${{{{\mathcal{L}}}}}_{{{{\rm{current}}}}}$ was then:

17 $${{{{{g}}}}}_{{{{\rm{current}}}}}=\frac{1}{| {{{{\mathcal{B}}}}}_{{{{\rm{current}}}}}| }\mathop{\sum}\limits_{({{{{x}}}},o)\in {{{{\mathcal{B}}}}}_{{{{\rm{current}}}}}}\;\frac{\delta {{{{\mathcal{L}}}}}^{{{{\rm{C}}}}}({{{{x}}}},o;{{{{\theta }}}})}{\delta {{{{\theta }}}}}$$

and the gradient of ${{{{\mathcal{L}}}}}_{{{{\rm{replay}}}}}$ was given by:

18 $${{{{{g}}}}}_{{{{\rm{replay}}}}}=\frac{1}{| {{{{\mathcal{B}}}}}_{{{{\rm{replay}}}}}| }\mathop{\sum}\limits_{({{{{x}}}},o)\in {{{{\mathcal{B}}}}}_{{{{\rm{replay}}}}}}\;\frac{\delta {{{{\mathcal{L}}}}}^{{{{\rm{C}}}}}({{{{x}}}},o;{{{{\theta }}}})}{\delta {{{{\theta }}}}}$$

The gradient g^*^ used to update the parameters was then given by:

19 $${{{{{g}}}}}^{* }=\left\{\begin{array}{ll}{{{{{g}}}}}_{{{{\rm{current}}}}}\quad &{{{\rm{if}}}}\,{{{{{{g}}}}}_{{{{\rm{current}}}}}}^{T}{{{{{g}}}}}_{{{{\rm{replay}}}}}\ge 0\\ {{{{{g}}}}}_{{{{\rm{current}}}}}-\frac{{{{{{{g}}}}}_{{{{\rm{current}}}}}}^{T}{{{{{g}}}}}_{{{{\rm{replay}}}}}}{\left({{{{{{g}}}}}_{{{{\rm{replay}}}}}}^{T}{{{{{g}}}}}_{{{{\rm{replay}}}}}+\gamma \right)}{{{{{g}}}}}_{{{{\rm{replay}}}}}\quad &{{{\rm{otherwise}}}},\end{array}\right.$$

with *γ* a small constant to ensure numerical stability. In A-GEM’s original formulation^54^ there was no *γ*-term, but we found that without it, performance was unstable. We used *γ* = 1 × 10^−7^.

#### DGR

With DGR^27^, two neural networks were sequentially trained on all contexts: a classifier, for which we used the base neural network, and a separate generative model.

For training of the classifier, as with ER and A-GEM, ${{{{\mathcal{L}}}}}_{{{{\rm{current}}}}}$ and ${{{{\mathcal{L}}}}}_{{{{\rm{replay}}}}}$ were the standard classification loss on the data of the current context and the replayed data, respectively. With DGR, the replayed data was obtained by sampling inputs from a copy of the generative model and labelling them as the most likely class predicted for those inputs by a copy of the classifier. The samples replayed during context *K* were generated by copies of the generator and classifier stored directly after finishing training on context *K* − 1. With task-incremental learning, each replayed sample was labelled and evaluated separately for all previous contexts and ${{{{\mathcal{L}}}}}_{{{{\rm{replay}}}}}$ was the average over those contexts.

As generative model a variational autoencoder (VAE; ref. ^72^) was used, which consisted of an encoder network *q*_*ϕ*_ that mapped an input-vector *x* to a vector of latent variables *z*, and a decoder network *p*_*ψ*_ that mapped those latent variables back to a reconstructed or decoded input-vector ${{{\hat{{x}}}}}$. The architecture of these two networks was kept similar to that of the base neural network: for Split MNIST, the encoder and the decoder were both fully connected networks with two hidden layers of 400 ReLU each; for Split CIFAR-100, the encoder consisted of the same five pre-trained convolutional layers as the base neural network followed by two fully connected layers with 2,000 ReLU units, and the decoder consisted of two fully connected layers with 2,000 ReLU followed by five deconvolutional (or transposed convolutional) layers^73^ that mirrored the convolutional layers and contained 128, 64, 32, 16 and 3 channels. The first four deconvolutional layers used a 4×4 kernel, a padding of 1 and a stride of 2 (that is, image size was doubled in each of those layers), while the final layer used a 3×3 kernel, a padding of 1 and a stride of 1 (that is, no upsampling). Batch-norm and ReLU non-linearities were used in all deconvolutional layers except for the last one. For both context sets, the VAE’s latent variable layer *z* had 100 Gaussian units. The prior over the latent variables was the standard normal distribution.

For a given input *x*, the loss function for training the parameters of the VAE was:

20 $${{{{\mathcal{L}}}}}^{{{{\rm{G}}}}}\left({{{{x}}}};{{{{\phi }}}},{{{{\psi }}}}\right)={{{{\mathcal{L}}}}}^{{{{\rm{latent}}}}}\left({{{{x}}}};{{{{\phi }}}}\right)+{{{{\mathcal{L}}}}}^{{{{\rm{recon}}}}}\left({{{{x}}}};{{{{\phi }}}},{{{{\psi }}}}\right)$$

The first term in equation (20), the ‘latent variable regularization term’, was given by:

21 $${{{{\mathcal{L}}}}}^{{{{\rm{latent}}}}}({{{{x}}}};{{{{\phi }}}})=\frac{1}{2}\mathop{\sum }\limits_{j=1}^{{N}_{{{{\rm{latent}}}}}}\left(1+\log ({{\sigma }_{j}^{({{{{x}}}})}}^{2})-{{\mu }_{j}^{({{{{x}}}})}}^{2}-{{\sigma }_{j}^{({{{{x}}}})}}^{2}\right)$$

with *N*_latent_ the number of latent variables, and ${\mu }_{j}^{({{{\boldsymbol{x}}}})}$ and ${\sigma }_{j}^{({{{{x}}}})}$ the *j*^th^ elements of *μ*^(*x*)^ and *σ*^(*x*)^, which were the outputs of the encoder network *q*_*ϕ*_ for input *x*. The second term in equation (20), the ‘reconstruction term’, was given by the squared error between the original and decoded pixel values:

22 $${{{{\mathcal{L}}}}}^{{{{\rm{recon}}}}}\left({{{{x}}}};{{{{\phi }}}},{{{{\psi }}}}\right)=\mathop{\sum }\limits_{p=1}^{{N}_{{{{\rm{pixels}}}}}}{\left({x}_{p}-{\tilde{x}}_{p}\right)}^{2}$$

whereby *x*_*p*_ was the value of the *p*^th^ pixel of the original input image *x* and ${\tilde{x}}_{p}$ was the value of the *p*^th^ pixel of the decoded image $\tilde{{{{{x}}}}}={p}_{{{{{\psi }}}}}\left({{{{{z}}}}}^{({{{{x}}}})}\right)$, with *z*^(*x*)^ = *μ*^(*x*)^ + *σ*^(*x*)^ × *ϵ* and *ϵ* sampled from ${{{\mathcal{N}}}}\left(0,{{{{I}}}}\right)$.

Training of the generative model was also done with generative replay, which was provided by its own copy stored after finishing training on the previous context. The loss terms of the current and replayed data were weighted similarly to the classifier:

23 $${{{{\mathcal{L}}}}}_{{{{\rm{total}}}}}^{{{{\rm{G}}}}}=\frac{1}{{N}_{{{{\rm{contexts}}}}\,{{{\rm{so}}}}\,{{{\rm{far}}}}}}{{{{\mathcal{L}}}}}_{{{{\rm{current}}}}}^{{{{\rm{G}}}}}+(1-\frac{1}{{N}_{{{{\rm{contexts}}}}\,{{{\rm{so}}}}\,{{{\rm{far}}}}}}){{{{\mathcal{L}}}}}_{{{{\rm{replay}}}}}^{{{{\rm{G}}}}}$$

#### BI-R

For the method BI-R, we followed the protocol as described in the original paper^28^. For Split CIFAR-100, all five of the proposed modifications relative to DGR were used: distillation, replay-through-feedback, conditional replay, gating based on internal context and internal replay. For Split MNIST, internal replay was not used, but the other four components were used. We did not combine BI-R with SI. The hyperparameter *X*, which controlled the proportion of hidden units in the decoder that was gated per class, was set based on a grid search (Supplementary Note 8).

Compared with ref. ^28^ there were two slight differences: (1) here we used a different set of pre-trained convolutional layers for each random seed, while ref. ^28^ always used the same pre-trained convolutional layers; and (2) in the class-incremental learning scenario, here we used a softmax layer with the output units of all classes always set to active, while ref. ^28^ used an ‘expanding head’ (that is, only the output units of classes seen so far were set to active).

### Template-based classification methods

Although for the context sets considered in this article, the template-based classification methods could, in theory, be used for all three continual learning scenarios, we considered them only for class-incremental learning. This was because, from an incremental-learning perspective, the specific benefit of template-based classification (that is, rephrasing a class-incremental learning problem as a task-incremental learning problem, see Supplementary Note 4) is only relevant in that scenario.

#### Generative classifier

For the generative classifier^55^, a separate VAE model was trained for each class to be learned. Training of these models was done as described above for DGR, except that no replay was used and each VAE was only trained on the examples from its own class. Each class-specific VAE was trained for either 1,000 iterations (Split MNIST) or 500 iterations (Split CIFAR-100), which meant that the total number of training iterations was the same as for the other methods. The mini-batch size was also the same: 128 for Split MNIST and 256 for Split CIFAR-100.

The architecture of the VAE models was chosen so that the total number of parameters of the generative classifier was similar to the number of parameters used by generative replay. For Split MNIST, the encoder and the decoder were both fully connected networks with two hidden layers of 85 ReLU units each and the latent variable layer had five units. For Split CIFAR-100, the pre-trained convolutional layers were used as a feature extractor, and the VAE models were trained on the extracted features rather than on the raw inputs (that is, the reconstruction loss was in the feature space instead of at the pixel level). The encoder and decoder both had one fully connected hidden layer with 85 ReLU and a latent variable layer with 20 units.

Classification was performed based on Bayes’ rule: a test sample was classified as the class under whose generative model it was estimated to be the most likely. That is, the output class label o^*^ predicted for an input *x* was given by:

24 $$o^* = \mathop{\rm{arg}\,{\rm{max}}}\limits_o\,p\left({{x}}|o\right)$$

whereby *p*(***x***∣*o*) was the likelihood of input *x* under the generative model of class *o*. These likelihoods were estimated using importance sampling^74^:

25 $$p({{{{x}}}}| o)=\frac{1}{S}\mathop{\sum }\limits_{s=1}^{S}\frac{f\left({{{{x}}}}\left|\,{{{{{\mu }}}}}_{{{{{{\psi }}}}}_{o}}^{\left({{{{{z}}}}}^{(s)}\right)},I\right.\right)f\left(\left.{{{{{z}}}}}^{(s)}\right|\,{{{{0}}}},I\right)}{f\left({{{{{z}}}}}^{(s)}\left|\,{{{{{\mu }}}}}_{{{{{{\phi }}}}}_{o}}^{({{{{x}}}})},{{{{{{\sigma }}}}}_{{{{{{\phi }}}}}_{o}}^{({{{{x}}}})}}^{2}I\right.\right)}$$

with ${{{{{\mu }}}}}_{{{{{{\phi }}}}}_{o}}^{({{{{x}}}})}$ and ${{{{{\sigma }}}}}_{{{{{{\phi }}}}}_{o}}^{({{{{x}}}})}$ the outputs of the encoder network for input *x*, ${{{{{\mu }}}}}_{{{{{{\psi }}}}}_{o}}^{\left({{{{z}}}}\right)}$ the output of the decoder network for input *z*, *S* the number of importance samples and *z*^(*s*)^ the *s*^th^ importance sample drawn from ${{{\mathcal{N}}}}\left({{{{{\mu }}}}}_{{{{{{\phi }}}}}_{o}}^{({{{{x}}}})},{{{{{{\sigma }}}}}_{{{{{{\phi }}}}}_{o}}^{({{{{x}}}})}}^{2}I\right)$. In this notation, $f\left({{{{x}}}}\left|\,{{{{\mu }}}},{{\Sigma }}\right.\right)$ indicates the probability density of *x* under the multivariate normal distribution with mean *μ* and covariance matrix Σ. Similar to ref. ^55^, we used *S* = 10,000 importance samples per likelihood estimation.

#### iCaRL

The method iCaRL (ref. ^25^) used a neural network for feature extraction and then performed classification based on a nearest-class-mean rule in that feature space, whereby the class means were calculated from stored data. To protect the feature extractor network from becoming unsuitable for previously learned contexts, iCaRL also replayed the stored data—as well as the inputs from the current context with a special form of distillation—during training of the feature extractor.

For the feature extractor we used the base neural network, except with the softmax output layer removed. We denote this feature extractor by *ψ*_*ϕ*_(.), with trainable parameters *ϕ*. These parameters were trained based on a binary classification/distillation loss. For this, during training only, a sigmoid output layer was appended to *ψ*_*ϕ*_. The resulting extended network outputs for any output class *o* ∈ {1, …, *N*_classes so far_} a binary probability whether input *x* belongs to it:

26 $${p}_{{{{{\theta }}}}}^{o}({{{{x}}}})=\frac{1}{1+{\mathrm{e}}^{-{{{{{w}}}}}_{o}^{T}{\psi }_{{{{{\phi }}}}}({{{{x}}}})}}$$

with ${{{{\theta }}}}=\left({{{{\phi }}}},{{{{{w}}}}}_{1},\ldots,{{{{{w}}}}}_{{N}_{{{{\rm{classes}\,{so}\,{far}}}}}}\right)$ a vector containing all the trainable parameters of iCaRL. Whenever a new output class *o* was encountered, new parameters *w*_*o*_ were added to *θ*.

In each context, the parameters in *θ* were trained on an extended dataset containing the current context’s training data as well as all stored data in the memory buffer. When training on context *K*, each input *x* with hard target *o* in this extended dataset was paired with a new target-vector ${{{\bar{{o}}}}}$ whose *j*^th^ element was given by:

27 $${\bar{o}}_{j}=\left\{\begin{array}{ll}{p}_{{\hat{{{{{\theta }}}}}}^{* }}^{j}\left({{{{x}}}}\right)\quad &{{{\rm{if}}}}\,{{{\rm{output}}}}\,{{{\rm{class}}}}\,j\,{{{\rm{in}}}}\,{{{\rm{context}}}}\,1,\ldots,K-1\\ {{\mathbb{1}}}_{\left\{o = j\right\}}\quad &{{{\rm{if}}}}\,{{{\rm{output}}}}\,{{{\rm{class}}}}\,j\,{{{\rm{in}}}}\,{{{\rm{context}}}}\,K\end{array}\right.$$

whereby ${\hat{{{{{\theta }}}}}}^{* }$ is the vector with parameter values at the end of training on context *K* − 1. The binary classification/distillation loss function for an input *x* labelled with such an ‘old-context-soft-target/new-context-hard-target’ vector ${{{\bar{{o}}}}}$ was then given by:

28 $${{{{\mathcal{L}}}}}_{{{{\rm{iCaRL}}}}}\left({{{{x}}}},{{{\bar{{o}}}}};{{{{\theta }}}}\right)=-\mathop{\sum }\limits_{j=1}^{{N}_{{{{\rm{classes}}}}\,{{{\rm{so}}}}\,{{{\rm{far}}}}}}\left[{\bar{o}}_{j}\log {p}_{{{{{\theta }}}}}^{j}({{{{x}}}})+\left(1-{\bar{o}}_{j}\right)\log \left(1-{p}_{{{{{\theta }}}}}^{j}({{{{x}}}})\right)\right]$$

After finishing training on a context, data to be added to the memory buffer were selected as follows. For each new output class *o*, iteratively *B* samples (or ‘exemplars’) were selected based on their extracted feature vectors according to a procedure referred to as ‘herding’. In each iteration, a new sample from output class *o* was selected such that the average feature vector over all selected examples was as close as possible to the average feature vector over all available examples of class *o*. Let ${{{{\mathcal{X}}}}}^{o}=\{{{{{{x}}}}}_{1},...,{{{{{x}}}}}_{{N}_{o}}\}$ be the set of all available examples of class *o* and let ${{{{{\mu }}}}}^{o}=\frac{1}{{N}_{o}}{\sum }_{{{{{x}}}}\in {{{{\mathcal{X}}}}}^{o}}{\psi }_{{{{{\phi }}}}}({{{{x}}}})$ be the average feature vector over set ${{{{\mathcal{X}}}}}^{o}$. The *n*^th^ exemplar (for *n* = 1, …, *m*) to be selected for output class *o* was then given by:

29 $${{p}}_{n}^{o} = \mathop{\rm{arg}\,{\rm{min}}}\limits_{{{x}}\,\in {\mathcal{X}}^o} \left\|{{\mu}}^o-\frac{1}{n}\left(\psi_{{{\phi}}}({{x}})+\sum\limits_{i=1}^{n-1}\psi_{{{\phi}}}({{p}}_{i}^{o})\right)\right\|$$

This resulted in ordered exemplar-sets ${{{{\mathcal{P}}}}}^{o}=\{{{{{{p}}}}}_{1}^{o},\ldots,{{{{{p}}}}}_{m}^{o}\}$ for each new output class *o* that were stored in the memory buffer.

Finally, classification was performed based on a nearest-class-mean rule in feature space, whereby the class means were calculated from the stored exemplars. For this, let ${{{{{\mu }}}}}_{o}=\frac{1}{\left|{{{{\mathcal{P}}}}}^{o}\right|}{\sum }_{{{{{p}}}}\in {{{{\mathcal{P}}}}}^{o}}{\psi }_{{{{{\phi }}}}}({{{{p}}}})$ for *o* = 1, …, *N*_classes so far_. The output class label o^*^ predicted for a new input *x* was then given by:

30 $$o^* = \mathop{\rm{arg}{\rm{min}}}\limits_{o=1,\ldots,N_{{\rm{classes}}\,{\rm{so}}\,{\rm{far}}}}\,\left\|\psi_{{{\phi}}}({{x}})-{{\mu}}_o\right\|$$

## Supplementary information

Supplementary Information Supplementary Notes 1–8 and references.

## Data Availability

All datasets used in this study are freely available online resources: http://yann.lecun.com/exdb/mnist/ (MNIST^66^) and https://www.cs.toronto.edu/~kriz/cifar.html (CIFAR-10 and CIFAR-100^67^).

## Code availability

Documented code that can be used to reproduce or build upon the reported experiments is available online under an MIT licence: https://github.com/GMvandeVen/continual-learning^75^.
