# Supplementary material for: Three types of incremental learning
Source: Nat Mach Intell. 2022 Dec 5;4(12):1185–97. doi: 10.1038/s42256-022-00568-3 (PMC9771807; doi:10.1038/s42256-022-00568-3)
Supplement: Supplementary file 1 — Supplementary Notes 1–8 and references. [file 42256_2022_568_MOESM1_ESM.pdf]

nature machine intelligence

Article https://doi.org/10.1038/s42256-022-00568-3

# Three types of incremental learning

In the format provided by the authors and unedited

## Table of contents


Supplementary Note 1: Further examples illustrating the three continual learning scenarios 2

Supplementary Note 2: An example beyond the academic continual learning setting 3

Supplementary Note 3: An example of a mixture of scenarios 6

Supplementary Note 4: Strategies for continual learning 7

Supplementary Note 5: Relevance for unsupervised and reinforcement learning 9

Supplementary Note 6: Other categorizations of continual learning 10

Supplementary Note 7: Different ways of using context identity information 11

Supplementary Note 8: Hyperparameters 12

Supplementary References 14

## Supplementary Note 1: Further examples illustrating the three continual learning scenarios

**Two-context example.** To make the difference between the three continual learning scenarios more intuitive, here we provide a simple example with two contexts (Table 1.1). In the first context, images of cats and chickens are shown, while the second context has images of dogs and ducks. After both contexts have been experienced, the question that is asked at test time can be formulated differently for each of the three scenarios. With class-incremental learning (Class-IL), because the algorithm must be able to distinguish between all classes, the question at test time is "Is it a cat, a chicken, a dog or a duck?". With domain-incremental learning (Domain-IL) the algorithm needs to decide whether an image belongs to a context's first category (i.e., a 'cat' or a 'dog') or to its second category (i.e., a 'chicken' or a 'duck'), but without knowing from which context the image is; the question at test time can therefore be formulated as "Is it a mammal or a bird?". With task-incremental learning (Task-IL), because context identity is known at test time, the question can be made more specific: depending on the context from which the test example comes, the question is either "Is it a cat or a chicken?" or "Is it a dog or a duck?".

| Context 1 | Context 2 |
|---|---|
| 'cats' & 'chickens' | 'dogs' & 'ducks' |

***Question at test time:***

| | |
|---|---|
| **Task-IL** | "Is it a cat or a chicken?" OR "Is it a dog or a duck?" |
| **Domain-IL** | "Is it a mammal or a bird?" |
| **Class-IL** | "Is it a cat, a chicken, a dog or a duck?" |

**Table 1.1 | A two-context example.**

**Permuted MNIST.** As another example, we illustrate the three continual learning scenarios on the popular 'Permuted MNIST' protocol[1]. With Permuted MNIST, every context involves classifying all ten MNIST-digits, but for each context a different permutation is applied to the pixels of each image (Fig. 1.1). Although it could be argued that Permuted MNIST is most naturally performed according to the domain-incremental learning scenario (e.g., refs.[1–6]), this benchmark can be performed according to the other scenarios too (Table 1.2). Indeed, examples can be found in the literature of Permuted MNIST being performed according to the task-incremental learning scenario[7,8] and the class-incremental learning scenario[9] as well.

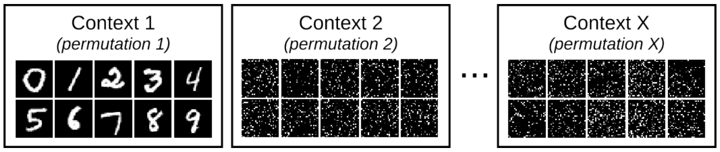


**Fig. 1.1 | Schematic of Permuted MNIST.**

| | |
|---|---|
| **Task-incremental learning** | Given the permutation, which digit is? |
| **Domain-incremental learning** | With the permutation unknown, which digit is? |
| **Class-incremental learning** | Which digit is it *and* which permutation is it? |

**Table 1.2 | Permuted MNIST according to the three scenarios.** Description of the type of choice an algorithm is expected to make when Permuted MNIST is performed according to each of the three scenarios.

## Supplementary Note 2: An example beyond the academic continual learning setting

Here we provide an example to illustrate the generalized versions of the three continual learning scenario. Similar as is done for the academic continual learning setting in the main text, the MNIST dataset is used to demonstrate how a given ‘task-free’ data stream[10–13] can be performed in three different ways. Moreover, using this example, we also perform an empirical evaluation to compare the generalized scenarios with each other and to test the efficacy of different computational strategies on each of them.

**Task-free version of Split MNIST.** We start by describing a task-free version of Split MNIST, in which there are no sharp boundaries between contexts and in which a context can be experienced more than once. We then illustrate how such a task-free continual learning experiment can be performed according to all three of the generalized versions of the continual learning scenarios defined in the main text.

The first step in setting up this continual learning experiment is defining the context set (i.e., the set of underlying distributions). We use the same context set as with the standard version of Split MNIST in the main text: the original MNIST-dataset is split up into five contexts in such a way that each contexts contains two digits (Fig. 2.1A). This means that the non-stationary aspect of the data in this experiment can be described as “the type of digit, in a pairwise manner”.

The second step to set up the experiment is defining the data stream (i.e., the set of experiences that are sequentially presented to the algorithm). In the academic continual learning setting, the data stream directly corresponds to the context set, as each experience simply contains all the training data of the corresponding context. Instead, here we follow the more general framework described by equation (1) in the main text, which states that the $i^{\text{th}}$ observation of experience $t$ is drawn from context $c$ with probability $p_c^{t,i}$. We use the following probabilities:

$$p_c^{t,i} = \begin{cases} x_c^t/1000 & \text{if } 0 < x_c^t \leq 1000 \\ 1 & \text{if } 1000 < x_c^t \leq 2000 \\ (3000-x_c^t)/1000 & \text{if } 2000 < x_c^t \leq 3000 \\ 0 & \text{otherwise} \end{cases} \qquad \textbf{(2.1)}$$

$$\text{with } \; x_c^t = (t-(500+(c-2)*2000)) \bmod 10{,}000$$

where $x \bmod y$ is the modulo operator that returns the remainder of the division of $x$ by $y$. The total number of experiences is $10,000$ and the number of observations per experience is 128. The resulting data stream has gradual transitions between contexts and the first context is revisited (Fig. 2.1B). Similar gradual transitions between contexts were used by refs.[4,9].

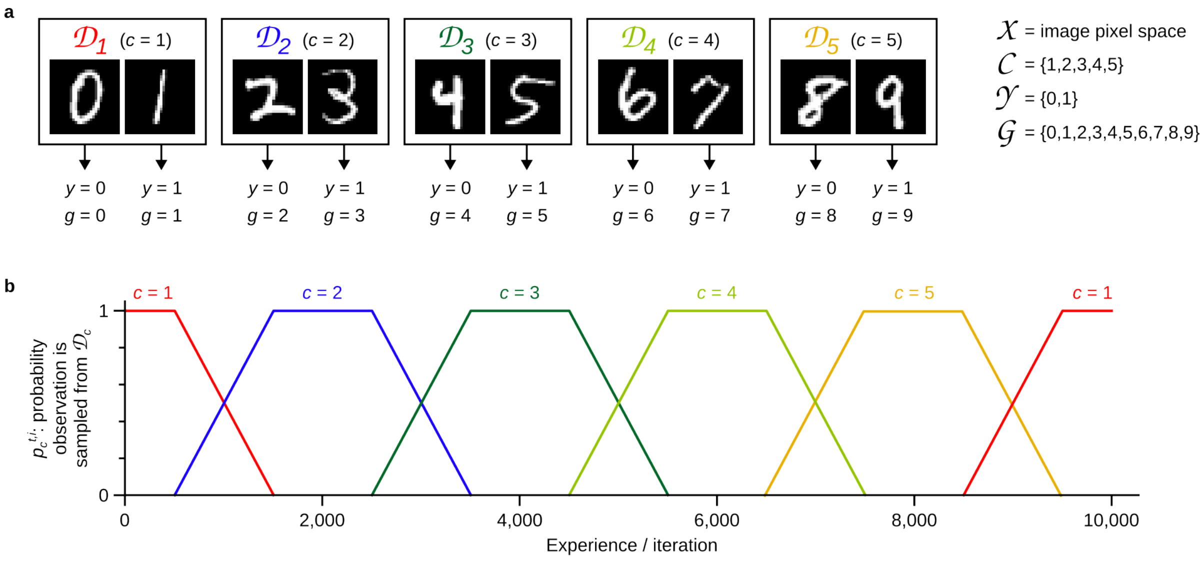


| | *Type of choice* | *Mapping to be learned* |
|---|---|---|
| **Generalized task-incremental learning** | Choice between two digits of same context (e.g., 0 or 1?) | $f: \mathcal{X} \times \mathcal{C} \rightarrow \mathcal{Y}$ |
| **Generalized domain-incremental learning** | Is the digit odd or even? | $f: \mathcal{X} \rightarrow \mathcal{Y}$ |
| **Generalized class-incremental learning** | Choice between all ten digits | $f: \mathcal{X} \rightarrow \mathcal{C} \times \mathcal{Y}$ or $f: \mathcal{X} \rightarrow \mathcal{G}$ |

**Fig. 2.1 | A task-free data stream can be performed according to each of the three generalized continual learning scenarios.** This figure illustrates three key components of a continual learning experiment: **a**, the *context set* specifies what aspect of the data changes over time; **b**, the *data stream* specifies how that aspect changes over time; and **c**, the *scenario* specifies how the aspect of the data that changes over time relates to the mapping that must be learned. Notation: $\mathcal{X}$ is the input space, $\mathcal{C}$ is the context space, $\mathcal{Y}$ is the within-context label space and $\mathcal{G}$ is the global label space. $\mathcal{D}_c$ is the underlying distribution of context $c$ and $p_c^{t,i}$ is the probability that the $i^{\text{th}}$ observation in experience $t$ is sampled from $\mathcal{D}_c$.

Importantly, as for the standard version of Split MNIST, this task-free version of Split MNIST can be performed in three different ways depending on how the mapping that must be learned relates to the context space: with generalized task-incremental learning the algorithm must learn the mapping $f\colon \mathcal{X}\times\mathcal{C}\rightarrow\mathcal{Y}$ (i.e., context labels are known at test time), with generalized domain-incremental learning the mapping to be learned is $f\colon \mathcal{X}\rightarrow\mathcal{Y}$, and with generalized class-incremental learning the mapping $f\colon \mathcal{X}\rightarrow\mathcal{C}\times\mathcal{Y}$ (or $f\colon \mathcal{X}\rightarrow\mathcal{G}$) must be learned (Fig. 2.1C). As in the academic setting, domain- and class-incremental learning can be distinguished by whether the expected output is the within-context label or the global label as well.

**Empirical comparison.** Next, using the task-free version of Split MNIST outlined above, we set out to perform an empirical comparison to test how well the different computational strategies work in each of the three generalized continual learning scenarios. However, not all methods that were used for the empirical comparisons in the main text could be applied in a task-free setting in a straight-forward manner, because some methods require context boundaries to perform certain consolidation operations (e.g., update the regularization term in the loss function or replace the copy of the model used for generating replay). A way around this is to instead perform these consolidation operations at regular intervals; in our experiments we did this after every 100 experiences. In other words, every 100 experiences were considered to be a new context. This approach resulted in feasible modified versions for the methods SI and LwF. On the other hand, for the methods EWC and FROMP this approach resulted in very high computational costs, while for the generative replay methods DGR and BI-R performance would likely fall sharply due to insufficient number of iterations between consolidation operations for the generative models to converge. These methods were therefore left out from the comparison. For the methods ER and A-GEM, rather than filling up the memory buffer with randomly selected samples after finishing training on a context, we instead used reservoir sampling[14,15]. For ER, the loss on the current experience and the loss on the replayed data were weighted equally: $\mathcal{L}_{\text{total}} = 0.5\mathcal{L}_{\text{current}} + 0.5\mathcal{L}_{\text{replay}}$. For the method iCaRL two modifications were made: reservoir sampling was used to fill the memory buffer (instead of the herding algorithm) and the feature extractor was trained using standard cross entropy loss with replay of samples from the memory buffer, using similar weighting as for ER above (instead of using the binary classification / distillation loss given by equation (28) in the main text). The approaches Separate Networks, XdG and Generative Classifier did not require modifications to work in this setting.

Similar as for the academic setting in the main text, here in this more flexible continual learning setting we found clear differences between the three generalized continual learning scenarios (Table 2.1). Importantly, we found qualitatively similar results as in the academic setting with regards to which computational strategies work well in which generalized scenarios. In particular, parameter and functional regularization achieved reasonable performance with generalized task-incremental learning, but they did not work well with generalized domain-incremental learning and they completely failed with class-incremental learning. Replay performed well in all three generalized scenarios, while template-based classification was able to match or surpass replay's performance with generalized class-incremental learning.

| **Strategy** | **Method** | **Budget** | **GM** | **Gen Task-IL** | **Gen Domain-IL** | **Gen Class-IL** |
|---|---|---|---|---|---|---|
| *Baselines* | *None – lower target* | | | *97.21 (± 0.53)* | *74.77 (± 1.67)* | *43.06 (± 0.67)* |
| | *Joint – upper target* | | | *99.59 (± 0.04)* | *98.60 (± 0.06)* | *98.13 (± 0.04)* |
| Context-specific components | Separate Networks | - | - | 98.75 (± 0.19) | - | - |
| | XdG | - | - | 98.29 (± 0.39) | - | - |
| Parameter regularization | Modified SI | - | - | 98.59 (± 0.44) | 78.42 (± 1.53) | 42.72 (± 0.92) |
| Functional regularization | Modified LwF | - | - | 99.40 (± 0.06) | 74.40 (± 1.52) | 41.42 (± 0.59) |
| Replay | ER | 1000 | - | 99.45 (± 0.04) | 95.72 (± 0.21) | 93.94 (± 0.19) |
| | A-GEM | 1000 | - | 99.33 (± 0.06) | 92.09 (± 0.68) | 86.71 (± 0.86) |
| Template-based classification | Generative Classifier | - | yes | - | - | 92.08 (± 0.20) |
| | Modified iCaRL | 1000 | - | - | - | 95.87 (± 0.09) |

**Table 2.1 | Results on the 'task-free' version of Split MNIST.** Reported is the final test accuracy (as %, averaged over all contexts) of all compared methods. Experiments were performed on the task-free data stream illustrated in Fig. 2.1. During training, context labels were available with the generalized versions of task- and class-incremental learning, but not in the generalized domain-incremental learning scenario. The column 'Budget' indicates the total number of examples that was allowed to be stored in a memory buffer; there was no separate memory per class. The column 'GM' indicates whether a generative model was learned, for which additional network capacity was used. Each experiment was performed 20 times with different random seeds, reported is the mean (± SEM) over these runs. Gen Task-IL: generalized task-incremental learning, Gen Domain-IL: generalized domain-incremental learning, Gen Class-IL: generalized class-incremental learning.

**Experimental details.** Unless stated otherwise above, the experimental settings for these experiments were similar as those that were used for the Split MNIST experiments in the main text. For example, the same optimizer, learning rate and base neural network architecture were used, and the set up of the output layer was analogous to the way it was set up in the academic setting. Similarly, the compared methods were implemented as described in the main text, except for the modifications discussed above. The hyperparameters for the methods XdG and modified SI were selected by a grid search (Table 2.2; see Supplementary Note 8 for a discussion of grid searches in continual learning).

| Method | Parameter | Explored range | Selected values<br>Gen Task-IL | <br>Gen Domain-IL | <br>Gen Class-IL |
|---|---|---|---|---|---|
| XdG | $X$ | $[0, 0.05, 0.1, ..., 0.9, 0.95]$ | 0.45 | - | - |
| Modified SI | $\gamma$ | $[0, 0.001, 0.01, ..., 10^6, 10^7]$ | 10 | 10 | 10 |

**Table 2.2** | **Hyperparameters.** Overview of the explored and selected hyperparameter values for the experiments with the task-free version of Split MNIST in Table 2.1.

## Supplementary Note 3: An example of a mixture of scenarios

Here we provide an example illustrating how the use of a multidimensional context space can result in continual learning problems that consist of multiple scenarios.

For this example we use the MNIST dataset with rotated images. The first dimension of the context space, denoted $\mathcal{C}_1$, divides the data into distinct contexts based on their class label, akin to the Split MNIST protocol (Fig. 3.1A). The second dimension of the context space, denoted $\mathcal{C}_2$, controls the amount by which a digit is rotated, akin to the Rotated MNIST protocol (Fig. 3.1B). These two dimensions of the context space can vary independently from each other: regardless of what digit an image contains, it can be rotated by all possible amounts. Importantly, each dimension of the context space could be performed according to a different scenario, which means that the resulting continual learning problem could be a mixture of two different scenarios (Table 3.1).

Real-world continual learning problems are often more complex and intertwined than this example. We believe that when solving such complex continual learning problems, it is often useful to break down the problem to its constituent parts by identifying what type(s) of continual learning must be done, as this will be an important determinant of what kind of continual learning strategies might be most appropriate.

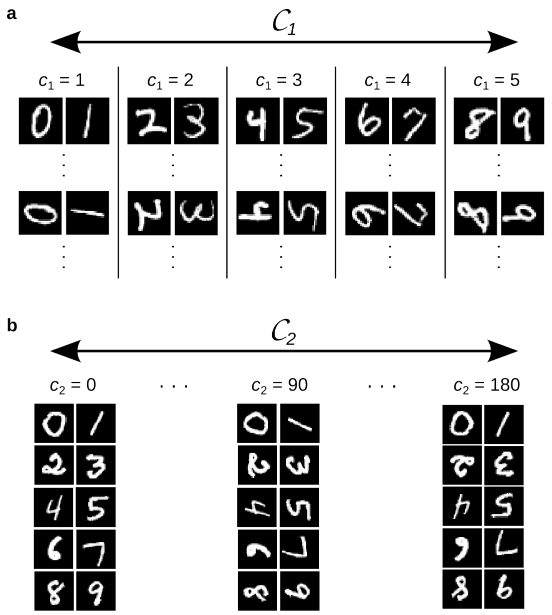


**Fig. 3.1 | A multidimensional context space.** Schematic illustrating a two-dimensional context space using the MNIST dataset with rotations. **a**, The first dimension $\mathcal{C}_1$ divides the digits based on their class label. **b**, The second dimension $\mathcal{C}_2$ controls the amount of rotation. Each dimension could be associated with a different scenario, allowing for the construction of continual learning problems that are mixtures of scenarios, see Table 3.1.

| | | $\mathcal{C}_1$ (split based on class labels) | | |
|---|---|---|---|---|
| | | **Task-IL** | **Domain-IL** | **Class-IL** |
| $\mathcal{C}_2$ (rotation) | **Task-IL** | Rotation is given, choice between 2 known digits | Rotation is given, choice between odd and even | Rotation is given, choice between all 10 digits |
| | **Domain-IL** | Rotation unknown, choice between 2 known digits | Rotation unknown, choice between odd and even | Rotation unknown, choice between all 10 digits |
| | **Class-IL** | Identify rotation + choice between 2 known digits | Identify rotation + choice between odd and even | Identify rotation + choice between all 10 digits |

**Table 3.1 | Mixtures of scenarios.** Description of the type of choice an algorithm is expected to make for each possible combination of scenarios for the two-dimensional context space of Fig. 3.1.

## Supplementary Note 4: Strategies for continual learning

Here we discuss and categorize different strategies for continual learning with deep neural networks. We do not aim to provide an extensive review of continual learning methods (for such reviews, see refs.[16–18]), but rather we focus on the underlying computational strategies. Many recent methods for continual learning combine multiple of these strategies.

**Context-specific components.** One way to reduce interference between contexts is through context-specific components. That is, to use certain parts of a network only for specific contexts (Fig. 3A in the main text). A common example of context-specific components is the multi-headed layout, which means that there is a separate output layer for each context[19,20]. Context-specificity, or modularity, can also be imposed on a network by gating its nodes or weights in a different way for each context. Such context-specific gates could be learned, for example using evolutionary algorithms[21,22], gradient descent[8] or Hebbian plasticity[23], or they could be defined randomly and *a priori*[7]. Another way to achieve context-specificity is to add new components, and thus to dynamically expand the network, when a new context is learned[24–26].

In the extreme version of this strategy, there is a completely separate network for each context. In this case, there is no forgetting at all. However, such a full segregation does not have good scaling properties and it precludes positive transfer between contexts. We believe that a specific case of this strategy, whereby the available computational resources (e.g., number of parameters) are divided over all contexts and a separate network is learned for each, is an important but often overlooked baseline for task-incremental learning problems, upon which successful task-incremental learning methods should be able to improve.

In principle, context-specific components can only be used with task-incremental learning, because context identity is required to select the correct context-specific components. However, when combined with an algorithm for identifying the context to which a sample belongs, this strategy could also be used with domain- or class-incremental learning[27–32]. In this regard it is important to point out that context identification is a class-incremental learning problem, as the mapping that must be learned is of the form $f\colon \mathcal{X} \to \mathcal{C}$.

**Parameter regularization.** Another popular strategy for continual learning is parameter regularization. To reduce forgetting, this strategy encourages neural network parameters important for past contexts not to change too much when learning new contexts (Fig. 3B in the main text). This strategy can be motivated from a Bayesian perspective, as parameter regularization methods can often be interpreted as performing sequential approximate Bayesian inference on the parameters of a neural network[2,33,34].

A common way to do parameter regularization is by adding a regularization term to the loss that penalizes changes to the network's parameters $\boldsymbol{\theta}$ weighted by an estimate of their importance for previous contexts (e.g., refs.[2,3,35–37]):

$$\mathcal{L}_{\text{total}} = \mathcal{L} + \left\|\boldsymbol{\theta} - \boldsymbol{\theta}^*\right\|_{\Sigma} \quad \textbf{(4.1)}$$

whereby $\mathcal{L}$ is the loss on the current context, $\boldsymbol{\theta}^*$ is the parameter vector relative to which changes are penalized (e.g., the value of $\boldsymbol{\theta}$ after finishing training on the last context), $\Sigma$ is an estimate of how important parameters are for previous contexts and $\|.\|_{\Sigma}$ is a weighted norm. Typically, a weighted $L^2$-norm is used, in which case the regularization term is given by $\|\boldsymbol{\theta} - \boldsymbol{\theta}^*\|_{\Sigma} = \frac{1}{2}(\boldsymbol{\theta} - \boldsymbol{\theta}^*)^T \Sigma (\boldsymbol{\theta} - \boldsymbol{\theta}^*)$. Another way to do parameter regularization is by projecting gradients into a subspace orthogonal to the one important for past contexts, to encourage parameter updates that do not interfere with previously stored information[38–40].

A critical aspect of parameter regularization is the estimation of the importance of the parameters for previous contexts. A popular approach is to use the Fisher Information matrix of the last context[2], as under certain assumptions it reflects how much small changes to the parameters would increase the loss. Usually a diagonal approximation of the Fisher Information is used, which assumes that all parameters are independent, but this assumption can be relaxed by using alternative approximations[37,40,41]. A drawback of the Fisher Information is that its computation can be costly. Several other parameter regularization methods have substantially lower computational overhead by instead estimating a per-parameter importance online during training[3,35].

Parameter regularization does not require storing observations and it can be used, at least in theory, for all three scenarios. Many parameter regularization methods assume discrete contexts and knowledge about context switches during training, as that is when the regularization term is typically updated, but this assumption is sometimes relaxed[4,10].

**Functional regularization.** An important issue with parameter regularization is that the behaviour of deep neural networks depends on its parameters in complex ways, which makes it challenging to accurately estimate the true importance of parameters for previous contexts. Rather than in parameter space, it might therefore be more effective to perform regularization directly in the function space of a neural network[42–44] (Fig. 3C in the main text). Functional regularization encourages the input-output mapping $f_{\boldsymbol{\theta}}$ of the neural network to change not too much at a particular set of inputs, which we refer to as the 'anchor points', when training on a new context:

$$\mathcal{L}_{\text{total}} = \mathcal{L} + \langle f_{\boldsymbol{\theta}}, f_{\boldsymbol{\theta}^*} \rangle_{\mathcal{A}} \quad \textbf{(4.2)}$$

whereby $\mathcal{L}$ is the loss for the current context, $f_{\boldsymbol{\theta}^*}$ is the input-output mapping relative to which changes are penalized (e.g., the input-output mapping of the network after finishing training on the last context) and $\mathcal{A} \subset \mathcal{X}$ is the set of anchor points at which the divergence between $f_{\boldsymbol{\theta}}$ and $f_{\boldsymbol{\theta}^*}$ is measured.

A critical aspect of functional regularization is the selection of anchor points. The optimal set of anchor points contains all inputs from the previous contexts, but using that set is computationally very costly and requires storing all those inputs. An alternative, proposed by Li and Hoiem[45], is to use the inputs from the current context and measure the divergence between $f_{\boldsymbol{\theta}}$ and $f_{\boldsymbol{\theta}^*}$ with a knowledge distillation loss[46]. Advantage is that this does not require storing observations, but there is no guarantee that the inputs from the current context are suitable anchor points. Another line of work formulates neural networks as Gaussian Processes[47], which allows for summarizing the input

distributions of previous contexts with inducing points[43] or memorable inputs[44], and for performing functional regularization in a Bayesian framework.

As for parameter regularization, most functional regularization methods can be used with all three continual learning scenarios. These methods typically assume context boundaries are known, but there is work relaxing this assumption[43].

**Replay.** Another strategy for continual learning, referred to as 'replay' or 'rehearsal', is to complement the training data of a new context with data representative of previous contexts[48] (Fig. 3D in the main text). With exact or experience replay, observations from previous contexts are stored in a memory buffer and revisited when training on new contexts[15,49–53]. Usually it is assumed that a limited amount of data can be stored, and an open research question is how to optimally select the samples to populate the memory buffer[52–55]. An alternative to storing observations is to learn a generative model to generate the data to be replayed[5,56–62]. An issue with such generative replay is that incrementally training a generative model can be challenging, especially when the data are complex[63,64]. A work-around can be to generate and replay latent features rather than raw inputs[5,61], although that usually requires pre-training of the lower, non-replayed layers of the network.

Typically, when replay is used, the objective is to jointly optimize the loss on the current and replayed data. An alternative approach, originally proposed by Lopez-Paz and Ranzato[65], is to use the loss on the replayed data as inequality constraints when optimizing for the current context. The idea is that the loss for previous contexts should not increase, but that it should be allowed to decrease.

Replay is similar to functional regularization in the sense that both strategies protect past knowledge by operating in the function space of a network, but a difference is that replay additionally allows for continued training on previous contexts. While the goal of regularization is to preserve what *was learned*, replay can be thought of as aiming to promote what *should have been learned*. However, the distinction between replay and functional regularization is sometimes blurred. For example, a common approach for generative replay is to learn a generative model for the inputs, but to then label the generated inputs based on predictions made for them by a copy of the network as it was after finishing training on the previous context[5,56,60,64]. This version of generative replay is therefore also a form of functional regularization.

Replay is suitable for all three continual learning scenarios. Moreover, there is a growing literature focused on developing replay-based methods for when there are no clear context boundaries[51–53,63,66–68]. An important concern with replay is its computational efficiency, since it involves constantly retraining on all previously seen contexts. Promisingly, it has been suggested that, rather than replaying everything, it might be sufficient to only replay items that are similar to the new data[69]. Moreover, it has been shown that because 'not forgetting' is easier than 'learning', replaying relatively small amounts of data can already be enough[5].

**Template-based classification.** Lastly we discuss classification using templates, which can be used as a strategy for class-incremental learning. With template-based classification, a template — which can also be thought of as a model or a representation — is learned for each class, after which classification decisions are made based on which template is most suitable for the sample to be classified (Fig. 3E in the main text). A key advantage is that this rephrases an often challenging class-incremental learning problem as a typically easier addressable task-incremental learning problem, whereby each 'task' is to learn a template for a specific class[70].

A popular approach, with roots in cognitive science, is to use 'prototypes' as class templates[71]. In deep learning, a prototype is usually the mean vector of a class in an embedding or metric space defined by a neural network[72,73]. Samples to be classified are then assigned to the class of the prototype to which they are closest (i.e., classification is performed based on a nearest-class-mean rule in the embedding space[74]). With class-incremental learning, if the embedding network is fixed, this approach can be implemented by storing a single prototype per class[75]. However, the embedding network might need to be updated to better separate newly encountered classes. To prevent prototypes from drifting when the embedding network evolves, several methods store a number of examples for each class to recompute or update the prototypes after changes to the embedding network[54,76]. An alternative that relaxes the need to store data is to estimate and correct prototypes' drift based on the drift observed for data of the current context[77,78].

Another example of template-based classification is generative classification[70]. In this case, the template learned for each class is a generative model, and the template's suitability for a test sample is measured by the likelihood of the sample under the generative model. Similar as with generative replay, when a suitable feature extractor is available, generative classification can be performed on latent features rather than on the raw inputs. Generative classification does not require storing observations, but making classification decisions can be computationally expensive. A more efficient alternative could be to use an energy-based model and to compute for each class an energy value rather than a likelihood[9].

## Supplementary Note 5: Relevance for unsupervised and reinforcement learning

The main focus of this article is on supervised learning, and classification in particular, but aspects of the three continual learning scenarios described here are also relevant for unsupervised and reinforcement learning. Firstly, with both types of learning, an important distinction is whether it is clear from what underlying context an input or a problem is: task-incremental (or context-aware) versus “not task-incremental” (or context-unaware). Secondly, when context identity is not provided, in principle it is also possible to make the distinction whether context identity must be inferred or not. However, context inference is either a supervised problem (if context labels are provided during training) or an unsupervised problem (if context labels are not provided during training).

More generally, the perspective of how the non-stationary aspect of the data relates to the mapping to be learned, might be useful to generate more fine-grained categorizations of continual learning problems involving unsupervised or reinforcement learning.

## Supplementary Note 6: Other categorizations of continual learning

A key contribution of this article is proposing a categorization of continual learning problems based on how the non-stationary aspect of the data relates to the mapping to be learned. We believe this particular categorization is useful, as underpinned by our experimental comparisons, but other relevant and often complementary categorizations can be made as well. For example, continual learning problems can also be categorized based on whether transitions between contexts are sharp or gradual [4,79,80], whether a context can be experienced multiple times [81–83], how the different contexts relate to each other (e.g., how similar are they?) [84,85] or how many observations there are in each experience (online versus batch-wise continual learning) [75,86].

Another way to categorize continual learning problems is through the lens of dataset shift formalization [87,88]. A first distinction here is between 'real concept drift', whereby the causal relation between the inputs and outputs changes, and 'virtual drift', whereby only the distribution from which is sampled changes [79,80,89]. Virtual drift has been further dissected into 'domain drift', where the input distribution from which is sampled changes in such a way that there is no change in the output distribution, and 'virtual concept drift', where there is a change in the output distribution [79]. Although motivated from a different perspective, these types of drift can be related to the three scenarios described here: real concept drift associates with task-incremental learning as often the algorithm must be informed about the causal change, while domain drift and virtual concept drift have intuitive links to respectively domain- and class-incremental learning.

Some other recently proposed categorizations also have similarities with the three scenarios. A common distinction in the continual learning literature is between methods evaluated with a 'multi-headed' or a 'single-headed' layout [19,20]. A multi-headed layout is linked to task-incremental learning as it requires context identity to be known, while a single-headed layout does not. However, an important difference is that the multi-headed versus single-headed distinction is tied to the architectural layout of a network's output layer (i.e., this is a distinction at the algorithmic or implementational level), while the three scenarios reflect the conditions under which a model is evaluated (i.e., our categorization is at the computational level). This is relevant because, for example, a multi-headed output layer is not the only way to use context identity information [7,8,22,24,25]. Another distinction in the literature is between benchmarks with 'new instances' (NI) versus with 'new classes' (NC) [90,91]. NI benchmarks typically correspond to domain-incremental learning and NC benchmarks to class-incremental learning, but this mapping is not exact, as for example either type of benchmark could also be performed according to the task-incremental learning scenario.

## Supplementary Note 7: Different ways of using context identity information

In the task-incremental learning scenario, the most common way to use context identity information is in the form of a multi-headed output layer (i.e., to have a separate output layer for each context). This is often a sensible way to use context identity information, but it is not the only way. Here, we show an example where context identity information is more efficiently used in another way.

We use the Permuted MNIST protocol (Fig. 1.1) with a sequence of 10 different permutations. We compare a selection of the methods that were included in the experiments reported in the main text. In the first set of experiments, denoted ***Multihead***, context identity information is used in the 'standard' way, meaning that all methods use a multi-headed output layer. In the second set of experiments, denoted ***Singlehead + XdG***, context identity information is instead used in the network's hidden layers, by combining each method with XdG. As can be seen from Table 7.1, for all compared methods, using context identity information in the network's hidden layers gives stronger performance than the use of a multi-headed output layer. For reference, we also report performances when context identity information is not used at all (***Singlehead***), which thus corresponds to the domain-incremental learning scenario.

| **Strategy** | **Method** | **Budget** | **GM** | **Task-IL** *Multihead* | **Task-IL** *Singlehead + XdG* | **Domain-IL** *Singlehead* |
|---|---|---|---|---|---|---|
| *Baselines* | *None* | | | *79.29 (± 0.43)* | *90.55 (± 0.45)* | *78.88 (± 0.84)* |
| | *Joint* | | | *97.94 (± 0.02)* | *97.89 (± 0.02)* | *97.91 (± 0.04)* |
| Parameter regularization | EWC | - | - | 94.83 (± 0.04) | 96.87 (± 0.04) | 94.30 (± 0.15) |
| | SI | - | - | 95.03 (± 0.19) | 96.71 (± 0.10) | 95.27 (± 0.10) |
| Functional regularization | LwF | - | - | 73.02 (± 0.69) | 85.77 (± 0.77) | 62.21 (± 1.59) |
| Replay | DGR | - | yes | 94.79 (± 0.06) | 95.17 (± 0.05) | 95.17 (± 0.09) |
| | ER | 10 | - | 93.63 (± 0.11) | 95.47 (± 0.08) | 93.62 (± 0.09) |

**Table 7.1 | Permuted MNIST, with different ways of using context identity information.** Reported is the final test accuracy (as %, averaged over all 10 contexts). The column 'Budget' indicates the number of examples per class that was allowed to be stored in a memory buffer. The column 'GM' indicates whether a generative model was learned, for which additional network capacity was used. Each experiment was performed 5 times with different random seeds, reported is the mean (± SEM) over these runs. Task-IL: task-incremental learning, Domain-IL: domain-incremental learning.

**Experimental details.** A sequence of ten contexts was used for the Permuted MNIST experiments. Context identity was provided during training. Each context contained all ten MNIST digits, with in every context a different random permutation applied to the pixels. Before being permuted, the original MNIST images were zero-padded to 32x32 pixels. No other pre-processing was performed. The standard training/test-split was used, resulting in 60,000 training and 10,000 test images per context.

The base neural network had 2 fully-connected hidden layers of 1000 ReLU units each and a softmax output layer. Training was done for 5000 iterations per context, using mini-batches of size 128 and the ADAM-optimizer ($\beta_1 = 0.9$, $\beta_2 = 0.999$) with learning rate 0.0001.

Details of the compared methods are described in the *Methods* section in the main text. For EWC, to reduce the computational costs, the diagonal elements of the Fisher Information matrix were computed using 1000 samples (i.e., the outer summation in equation (5) in the main text was over 1000 randomly selected samples rather than over the entire training set). For DGR, the generative model was a variational autoencoder with the encoder and decoder network both containing 2 fully-connected hidden layers of 1000 ReLU units each. The latent variable layer had 100 Gaussian units.

With XdG, always 60% of the network's hidden units were masked in each context. This percentage was selected based on a grid search in which XdG was used with the baseline *None*, and the outcome of this grid search was then also used for XdG in combination with the other methods. For EWC and SI, separate grid searches were performed to select the value of their hyperparameters (Table 7.2). See Supplementary Note 8 for a discussion of grid searches in continual learning.

| **Method** | **Parameter** | **Explored range** | **Selected values** *Multihead* | **Selected values** *Singlehead + XdG* | **Selected values** *Singlehead* |
|---|---|---|---|---|---|
| EWC | $\lambda$ | $[0, 1, 10, ..., 10^6, 10^7]$ | 100 | 100 | 100 |
| SI | $\gamma$ | $[0, 0.01, 0.1, ..., 10^4, 10^5]$ | 10 | 10 | 10 |

**Table 7.2 | Hyperparameters.** Overview of the explored and selected hyperparameter values for the experiments with the Permuted MNIST protocol in Table 7.1.

## Supplementary Note 8: Hyperparameters

Several of the in this article compared continual learning methods have a hyperparameter. In deep learning, the typical way of setting the value of hyperparameters is by training models on the training set for a range of hyperparameter-values, and selecting those that result in the best performance on a separate validation set. This strategy has been adapted to the continual learning setting as training models on the full data stream with different hyperparameter-values using only every context's training data, and comparing their overall performances using separate validation sets (or sometimes the test sets) for each context. However, we would like to stress that this means that these hyperparameters are set (or learned) based on an evaluation using data from all contexts, which violates the continual learning principle of only being allowed to access each context's training data in the order specified by the data stream. Although it is tempting to think that it is acceptable to relax this principle for contexts' validation data, we argue here that it is not. A clear example of how using each context's validation data continuously throughout an incremental training protocol can lead to an unfair advantage is provided by ref.[92], in which after finishing training on each context a 'bias-removal parameter' is computed that optimizes performance on the validation sets of all contexts seen so far (see their section 3.3). Although the hyperparameters of the methods compared here are less influential than those in the above report, we believe that it is important to realize this issue associated with traditional grid searches in a continual learning setting and that at a minimum influential hyperparameters should be avoided in methods for continual learning.

Nevertheless, to give all methods the best possible chance, and to explore how influential the hyperparameters are, we did perform grid searches to set the values of hyperparameters (see Figures 8.1 and 8.2). Given the issue discussed above, we believe using validation sets for these grid searches has the risk of being misleading, and we evaluated the performances of all hyperparameters using the contexts' test sets. For each grid search, all experiments were run once, after which 10 or 20 new runs were executed using the selected hyperparameter-values to obtain the results in Tables 2 and 3 in the main text.

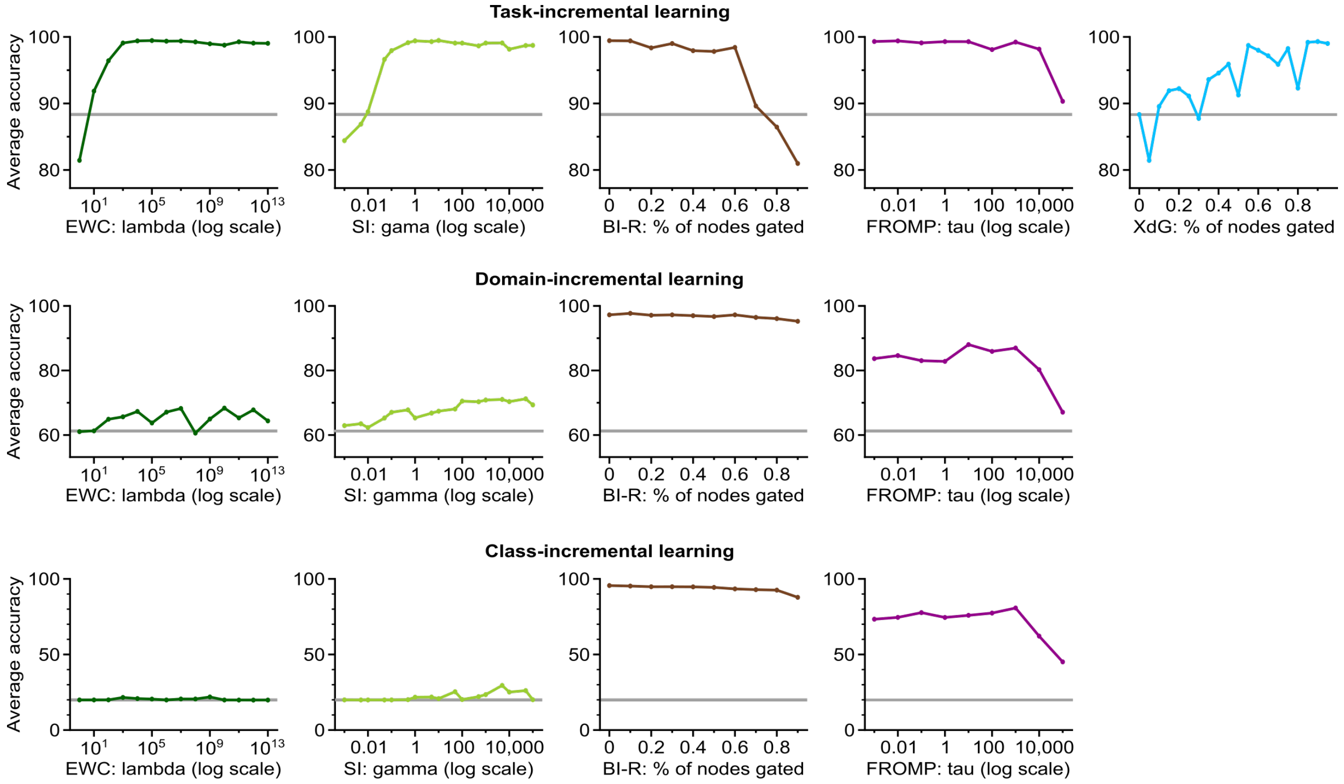


**Fig. 8.1 | Grid searches for Split MNIST.** Shown is the average test set accuracy (as %, over all 5 contexts) for the hyperparameter-values tested for each method. The horizontal grey line indicates the performance of the 'None' baseline.

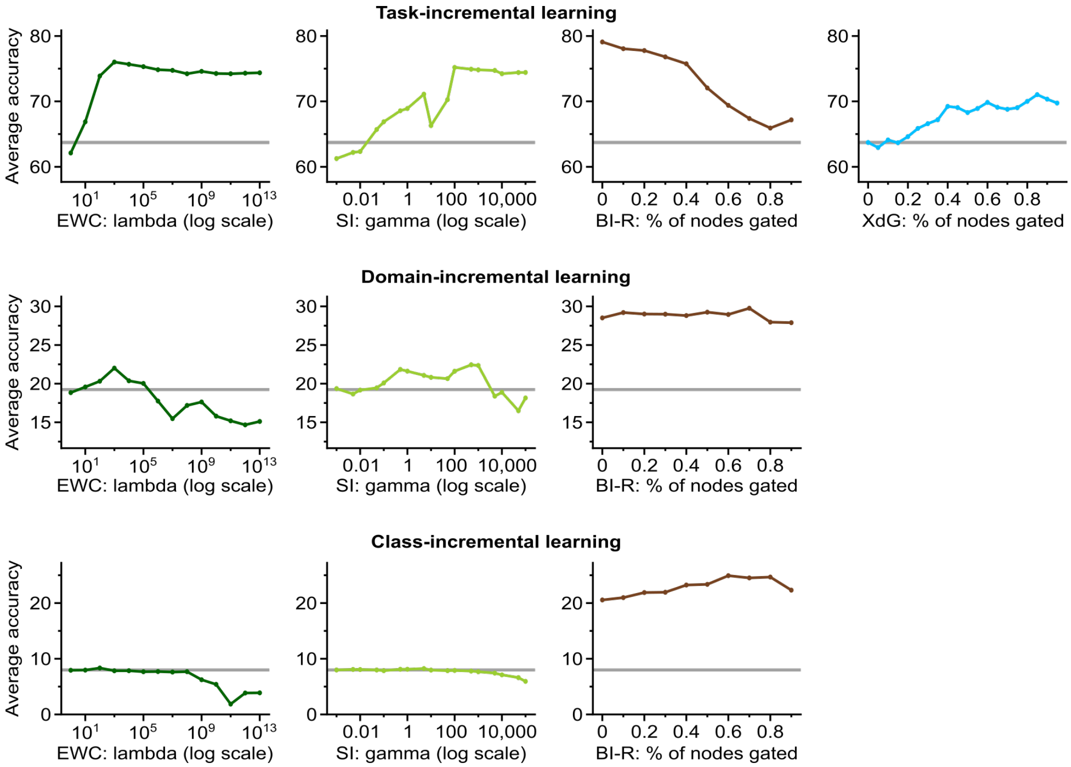


**Fig. 8.2 | Grid searches for Split CIFAR-100.** Shown is the average test set accuracy (as %, over all 10 contexts) for the hyperparameter-values tested for each method. The horizontal grey line indicates the performance of the 'None' baseline.

## Supplementary References


1. Goodfellow, I. J., Mirza, M., Xiao, D., Courville, A. & Bengio, Y. An empirical investigation of catastrophic forgetting in gradient-based neural networks. Preprint at https://arxiv.org/abs/1312.6211 (2013).
2. Kirkpatrick, J. *et al.* Overcoming catastrophic forgetting in neural networks. *Proceedings of the national academy of sciences* **114**, 3521–3526 (2017).
3. Zenke, F., Poole, B. & Ganguli, S. Continual learning through synaptic intelligence. In *International Conference on Machine Learning*, 3987–3995 (PMLR, 2017).
4. Zeno, C., Golan, I., Hoffer, E. & Soudry, D. Task agnostic continual learning using online variational bayes. Preprint at https://arxiv.org/abs/1803.10123v3 (2019).
5. van de Ven, G. M., Siegelmann, H. T. & Tolias, A. S. Brain-inspired replay for continual learning with artificial neural networks. *Nature Communications* **11**, 4069 (2020).
6. Wang, J., Sezener, E., Budden, D., Hutter, M. & Veness, J. A combinatorial perspective on transfer learning. *Advances in Neural Information Processing Systems* **33** (2020).
7. Masse, N. Y., Grant, G. D. & Freedman, D. J. Alleviating catastrophic forgetting using context-dependent gating and synaptic stabilization. *Proceedings of the national academy of sciences* E10467–E10475 (2018).
8. Serra, J., Suris, D., Miron, M. & Karatzoglou, A. Overcoming catastrophic forgetting with hard attention to the task. In *International Conference on Machine Learning*, 4548–4557 (PMLR, 2018).
9. Li, S., Du, Y., van de Ven, G. M. & Mordatch, I. Energy-based models for continual learning. Preprint at https://arxiv.org/abs/2011.12216 (2020).
10. Aljundi, R., Kelchtermans, K. & Tuytelaars, T. Task-free continual learning. In *Proceedings of the IEEE/CVF Conference on Computer Vision and Pattern Recognition*, 11254–11263 (2019).
11. Lee, S., Ha, J., Zhang, D. & Kim, G. A neural dirichlet process mixture model for task-free continual learning. In *International Conference on Learning Representations* (2020). URL https://openreview.net/forum?id=SJxSOJStPr.
12. Jin, X., Sadhu, A., Du, J. & Ren, X. Gradient-based editing of memory examples for online task-free continual learning. In *Advances in Neural Information Processing Systems*, vol. 34, 29193–29205 (2021).
13. Shanahan, M., Kaplanis, C. & Mitrović, J. Encoders and ensembles for task-free continual learning. Preprint at https://arxiv.org/abs/2105.13327 (2021).
14. Vitter, J. S. Random sampling with a reservoir. *ACM Transactions on Mathematical Software (TOMS)* **11**, 37–57 (1985).
15. Riemer, M. *et al.* Learning to learn without forgetting by maximizing transfer and minimizing interference. In *International Conference on Learning Representations* (2019). URL https://openreview.net/forum?id=B1gTShAct7.
16. Belouadah, E., Popescu, A. & Kanellos, I. A comprehensive study of class incremental learning algorithms for visual tasks. *Neural Networks* **135**, 38–54 (2021).
17. Masana, M. *et al.* Class-incremental learning: survey and performance evaluation. Preprint at https://arxiv.org/abs/2010.15277 (2020).
18. De Lange, M. *et al.* A continual learning survey: Defying forgetting in classification tasks. *IEEE Transactions on Pattern Analysis and Machine Intelligence* **44**, 3366–3385 (2021).
19. Farquhar, S. & Gal, Y. Towards robust evaluations of continual learning. Preprint at https://arxiv.org/abs/1805.09733 (2018).
20. Chaudhry, A., Dokania, P. K., Ajanthan, T. & Torr, P. H. Riemannian walk for incremental learning: Understanding forgetting and intransigence. In *Proceedings of the European Conference on Computer Vision (ECCV)*, 532–547 (2018).
21. Ellefsen, K. O., Mouret, J.-B. & Clune, J. Neural modularity helps organisms evolve to learn new skills without forgetting old skills. *PLoS computational biology* **11**, e1004128 (2015).
22. Fernando, C. *et al.* Pathnet: Evolution channels gradient descent in super neural networks. Preprint at https://arxiv.org/abs/1701.08734 (2017).
23. Flesch, T., Nagy, D. G., Saxe, A. & Summerfield, C. Modelling continual learning in humans with hebbian context gating and exponentially decaying task signals. Preprint at https://arxiv.org/abs/2203.11560 (2022).
24. Rusu, A. A. *et al.* Progressive neural networks. Preprint at https://arxiv.org/abs/1606.04671 (2016).
25. Yoon, J., Yang, E., Lee, J. & Hwang, S. J. Lifelong learning with dynamically expandable networks. In *International Conference on Learning Representations* (2018). URL https://openreview.net/forum?id=Sk7KsfW0-.
26. Vogelstein, J. T. *et al.* Representation ensembling for synergistic lifelong learning with quasilinear complexity. Preprint at https://arxiv.org/abs/2004.12908 (2020).
27. Aljundi, R., Chakravarty, P. & Tuytelaars, T. Expert gate: Lifelong learning with a network of experts. In *Proceedings of the IEEE Conference on Computer Vision and Pattern Recognition*, 3366–3375 (2017).
28. von Oswald, J., Henning, C., Sacramento, J. & Grewe, B. F. Continual learning with hypernetworks. In *International Conference on Learning Representations* (2020). URL https://openreview.net/forum?id=SJgwNerKvB.
29. Wortsman, M. *et al.* Supermasks in superposition. In *Advances in Neural Information Processing Systems*, vol. 33, 15173–15184 (2020).
30. Henning, C. *et al.* Posterior meta-replay for continual learning. In *Advances in Neural Information Processing Systems*, vol. 34, 14135–14149 (2021).
31. Verma, V. K., Liang, K. J., Mehta, N., Rai, P. & Carin, L. Efficient feature transformations for discriminative and generative continual learning. In *Proceedings of the IEEE/CVF Conference on Computer Vision and Pattern Recognition*, 13865–13875 (2021).
32. Heald, J. B., Lengyel, M. & Wolpert, D. M. Contextual inference underlies the learning of sensorimotor repertoires. *Nature* **600**, 489–493 (2021).
33. Nguyen, C. V., Li, Y., Bui, T. D. & Turner, R. E. Variational continual learning. In *International Conference on Learning Representations* (2018). URL https://openreview.net/forum?id=BkQqq0gRb.
34. Farquhar, S. & Gal, Y. A unifying bayesian view of continual learning. Preprint at https://arxiv.org/abs/1902.06494 (2019).
35. Aljundi, R., Babiloni, F., Elhoseiny, M., Rohrbach, M. & Tuytelaars, T. Memory aware synapses: Learning what (not) to forget. In *Proceedings of the European Conference on Computer Vision (ECCV)*, 139–154 (2018).
36. Schwarz, J. *et al.* Progress & compress: A scalable framework for continual learning. In *International Conference on Machine Learning*, 4528–4537 (PMLR, 2018).
37. Ritter, H., Botev, A. & Barber, D. Online structured laplace approximations for overcoming catastrophic forgetting. In *Advances in Neural Information Processing Systems)*, vol. 31 (2018).
38. Zeng, G., Chen, Y., Cui, B. & Yu, S. Continual learning of context-dependent processing in neural networks. *Nature Machine Intelligence* **1**, 364–372 (2019).
39. Duncker, L., Driscoll, L., Shenoy, K. V., Sahani, M. & Sussillo, D. Organizing recurrent network dynamics by task-computation to enable continual learning. In *Advances in Neural Information Processing Systems*, vol. 33, 14387–14397 (2020).
40. Kao, T.-C., Jensen, K., van de Ven, G., Bernacchia, A. & Hennequin, G. Natural continual learning: success is a journey, not (just) a destination. In *Advances in Neural Information Processing Systems*, vol. 34, 28067–28079 (2021).
41. Martens, J. & Grosse, R. Optimizing neural networks with kronecker-factored approximate curvature. In *International conference on machine learning*, 2408–2417 (PMLR, 2015).
42. Benjamin, A., Rolnick, D. & Kording, K. Measuring and regularizing networks in function space. In *International Conference on Learning Representations* (2019). URL https://openreview.net/forum?id=SkMwpiR9Y7.
43. Titsias, M. K., Schwarz, J., Matthews, A. G. d. G., Pascanu, R. & Teh, Y. W. Functional regularisation for continual learning with gaussian processes. In *International Conference on Learning Representations* (2020). URL https://openreview.net/forum?id=HkxCzeHFDB.
44. Pan, P. *et al.* Continual deep learning by functional regularisation of memorable past. In *Advances in Neural Information Processing Systems*, vol. 33, 4453–4464 (2020).
45. Li, Z. & Hoiem, D. Learning without forgetting. *IEEE Transactions on Pattern Analysis and Machine Intelligence* **40**, 2935–2947 (2017).
46. Hinton, G., Vinyals, O. & Dean, J. Distilling the knowledge in a neural network. Preprint at https://arxiv.org/abs/1503.02531 (2015).
47. Khan, M. E., Immer, A., Abedi, E. & Korzepa, M. Approximate inference turns deep networks into gaussian processes. In *Advances in Neural Information Processing Systems*, vol. 32 (2019).
48. Robins, A. Catastrophic forgetting, rehearsal and pseudorehearsal. *Connection Science* **7**, 123–146 (1995).
49. Rolnick, D., Ahuja, A., Schwarz, J., Lillicrap, T. & Wayne, G. Experience replay for continual learning. In *Advances in Neural Information Processing Systems*, vol. 32 (2019).
50. Buzzega, P., Boschini, M., Porrello, A. & Calderara, S. Rethinking experience replay: a bag of tricks for continual learning. In *International Conference on Pattern Recognition*, vol. 25, 2180–2187 (2021).
51. Pellegrini, L., Graffieti, G., Lomonaco, V. & Maltoni, D. Latent replay for real-time continual learning. In *IEEE/RSJ International Conference on Intelligent Robots and Systems*, 10203–10209 (IEEE, 2020).
52. Hayes, T. L., Cahill, N. D. & Kanan, C. Memory efficient experience replay for streaming learning. In *International Conference on Robotics and Automation*, 9769–9776 (2019).
53. Chaudhry, A. *et al.* On tiny episodic memories in continual learning. Preprint at https://arxiv.org/abs/1902.10486 (2019).
54. Rebuffi, S.-A., Kolesnikov, A., Sperl, G. & Lampert, C. H. icarl: Incremental classifier and representation learning. In *Proceedings of the IEEE Conference on Computer Vision and Pattern Recognition*, 2001–2010 (2017).
55. Mundt, M., Hong, Y. W., Pliushch, I. & Ramesh, V. A wholistic view of continual learning with deep neural networks: Forgotten lessons and the bridge to active and open world learning. Preprint at https://arxiv.org/abs/2009.01797 (2020).
56. Shin, H., Lee, J. K., Kim, J. & Kim, J. Continual learning with deep generative replay. In *Advances in Neural Information Processing Systems*, vol. 30, 2994–3003 (2017).
57. Mocanu, D. C., Vega, M. T., Eaton, E., Stone, P. & Liotta, A. Online contrastive divergence with generative replay: Experience replay without storing data. Preprint at https://arxiv.org/abs/1610.05555 (2016).
58. Wu, C. *et al.* Memory replay gans: learning to generate images from new categories without forgetting. In *Advances in Neural Information Processing Systems*, vol. 31, 5966–5976 (2018).
59. Rao, D. *et al.* Continual unsupervised representation learning. In *Advances in Neural Information Processing Systems*, vol. 32, 7647–7657 (2019).
60. Lesort, T., Gepperth, A., Stoian, A. & Filliat, D. Marginal replay vs conditional replay for continual learning. In *International Conference on Artificial Neural Networks*, 466–480 (Springer, 2019).
61. Liu, X. *et al.* Generative feature replay for class-incremental learning. In *Proceedings of the IEEE/CVF Conference on Computer Vision and Pattern Recognition (CVPR) Workshops*, 226–227

(2020).
62. Cong, Y., Zhao, M., Li, J., Wang, S. & Carin, L. Gan memory with no forgetting. In *Advances in Neural Information Processing Systems*, vol. 33, 16481–16494 (2020).
63. Aljundi, R. *et al.* Online continual learning with maximally interfered retrieval. In *Advances in Neural Information Processing Systems*, vol. 32 (2019).
64. Lesort, T., Caselles-Dupré, H., Garcia-Ortiz, M., Stoian, A. & Filliat, D. Generative models from the perspective of continual learning. In *International Joint Conference on Neural Networks*, 1–8 (IEEE, 2019).
65. Lopez-Paz, D. & Ranzato, M. Gradient episodic memory for continual learning. In *Advances in Neural Information Processing Systems*, vol. 30, 6470–6479 (2017).
66. Shim, D. *et al.* Online class-incremental continual learning with adversarial shapley value. In *Proceedings of the AAAI Conference on Artificial Intelligence*, vol. 35, 9630–9638 (2021).
67. Hayes, T. L. & Kanan, C. Selective replay enhances learning in online continual analogical reasoning. In *Proceedings of the IEEE/CVF Conference on Computer Vision and Pattern Recognition*, 3502–3512 (2021).
68. Buzzega, P., Boschini, M., Porrello, A., Abati, D. & Calderara, S. Dark experience for general continual learning: a strong, simple baseline. In *Advances in Neural Information Processing Systems*, vol. 33, 15920–15930 (2020).
69. McClelland, J. L., McNaughton, B. L. & Lampinen, A. K. Integration of new information in memory: new insights from a complementary learning systems perspective. *Philosophical Transactions of the Royal Society B* **375**, 20190637 (2020).
70. van de Ven, G. M., Li, Z. & Tolias, A. S. Class-incremental learning with generative classifiers. In *Proceedings of the IEEE/CVF Conference on Computer Vision and Pattern Recognition (CVPR) Workshops*, 3611–3620 (2021).
71. Nosofsky, R. M. Attention, similarity, and the identification–categorization relationship. *Journal of experimental psychology: General* **115**, 39 (1986).
72. Snell, J., Swersky, K. & Zemel, R. Prototypical networks for few-shot learning. In *Advances Neural Information Processing Systems*, vol. 30, 4080–4090 (2017).
73. Yang, H.-M., Zhang, X.-Y., Yin, F. & Liu, C.-L. Robust classification with convolutional prototype learning. In *Proceedings of the IEEE Conference on Computer Vision and Pattern Recognition*, 3474–3482 (2018).
74. Mensink, T., Verbeek, J., Perronnin, F. & Csurka, G. Metric learning for large scale image classification: Generalizing to new classes at near-zero cost. In *European Conference on Computer Vision*, 488–501 (Springer, 2012).
75. Hayes, T. L. & Kanan, C. Lifelong machine learning with deep streaming linear discriminant analysis. In *Proceedings of the IEEE/CVF Conference on Computer Vision and Pattern Recognition (CVPR) Workshops*, 220–221 (2020).
76. De Lange, M. & Tuytelaars, T. Continual prototype evolution: Learning online from non-stationary data streams. In *Proceedings of the IEEE/CVF International Conference on Computer Vision*, 8250–8259 (2021).
77. Yu, L. *et al.* Semantic drift compensation for class-incremental learning. In *Proceedings of the IEEE/CVF Conference on Computer Vision and Pattern Recognition Workshops*, 6982–6991 (2020).
78. Wei, K., Deng, C., Yang, X. & Li, M. Incremental embedding learning via zero-shot translation. In *Proceedings of the AAAI Conference on Artificial Intelligence*, vol. 35, 10254–10262 (2021).
79. Lesort, T., Caccia, M. & Rish, I. Understanding continual learning settings with data distribution drift analysis. Preprint at https://arxiv.org/abs/2104.01678 (2021).
80. Gepperth, A. & Hammer, B. Incremental learning algorithms and applications. In *European symposium on artificial neural networks (ESANN)* (2016).
81. Stojanov, S. *et al.* Incremental object learning from contiguous views. In *Proceedings of the IEEE/CVF Conference on Computer Vision and Pattern Recognition*, 8777–8786 (2019).
82. Caccia, L., Belilovsky, E., Caccia, M. & Pineau, J. Online learned continual compression with adaptive quantization modules. In *International Conference on Machine Learning*, 1240–1250 (PMLR, 2020).
83. Cossu, A. *et al.* Is class-incremental enough for continual learning? *Frontiers in Artificial Intelligence* **5**, 829842 (2022).
84. Ke, Z., Liu, B. & Huang, X. Continual learning of a mixed sequence of similar and dissimilar tasks. In *Advances in Neural Information Processing Systems*, vol. 33, 18493–18504 (2020).
85. Johnson, E. C. *et al.* L2explorer: A lifelong reinforcement learning assessment environment. Preprint at https://arxiv.org/abs/2203.07454 (2022).
86. Mai, Z. *et al.* Online continual learning in image classification: An empirical survey. *Neurocomputing* **469**, 28–51 (2022).
87. Quiñonero-Candela, J., Sugiyama, M., Schwaighofer, A. & Lawrence, N. D. *Dataset shift in machine learning* (Mit Press, 2008).
88. Moreno-Torres, J. G., Raeder, T., Alaiz-Rodríguez, R., Chawla, N. V. & Herrera, F. A unifying view on dataset shift in classification. *Pattern recognition* **45**, 521–530 (2012).
89. Gama, J., Žliobaitė, I., Bifet, A., Pechenizkiy, M. & Bouchachia, A. A survey on concept drift adaptation. *ACM computing surveys (CSUR)* **46**, 1–37 (2014).
90. Maltoni, D. & Lomonaco, V. Continuous learning in single-incremental-task scenarios. *Neural Networks* **116**, 56–73 (2019).
91. Lesort, T. *et al.* Continual learning for robotics: Definition, framework, learning strategies, opportunities and challenges. *Information fusion* **58**, 52–68 (2020).
92. Wu, Y. *et al.* Incremental classifier learning with generative adversarial networks. Preprint at https://arxiv.org/abs/1802.00853 (2018).
